# *Mycobacterium tuberculosis*: Pathogenesis and therapeutic targets

**DOI:** 10.1002/mco2.353

**Published:** 2023-09-04

**Authors:** Jiaxing Yang, Laiying Zhang, Wenliang Qiao, Youfu Luo

**Affiliations:** ^1^ Center of Infectious Diseases and State Key Laboratory of Biotherapy, West China Hospital Sichuan University Chengdu China; ^2^ Department of Thoracic Surgery, West China Hospital Sichuan University Chengdu Sichuan China; ^3^ Lung Cancer Center, West China Hospital Sichuan University Chengdu Sichuan China

**Keywords:** biological targets, host‐directed therapy, inhibitors, modulaters, *Mycobacterium tuberculosis*, pathogenesis

## Abstract

Tuberculosis (TB) remains a significant public health concern in the 21st century, especially due to drug resistance, coinfection with diseases like immunodeficiency syndrome (AIDS) and coronavirus disease 2019, and the lengthy and costly treatment protocols. In this review, we summarize the pathogenesis of TB infection, therapeutic targets, and corresponding modulators, including first‐line medications, current clinical trial drugs and molecules in preclinical assessment. Understanding the mechanisms of *Mycobacterium tuberculosis* (*Mtb*) infection and important biological targets can lead to innovative treatments. While most antitubercular agents target pathogen‐related processes, host‐directed therapy (HDT) modalities addressing immune defense, survival mechanisms, and immunopathology also hold promise. *Mtb*’s adaptation to the human host involves manipulating host cellular mechanisms, and HDT aims to disrupt this manipulation to enhance treatment effectiveness. Our review provides valuable insights for future anti‐TB drug development efforts.

## Graphical abstract

In this review, we summarize the pathogenesis of TB infection, therapeutic targets, and corresponding modulators, including first‐line medications, current clinical trial drugs and molecules in preclinical assessment. Our review provides valuable insights for future antituberculosis drug development efforts.

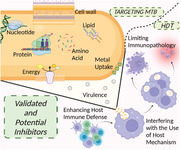

## 1 INTRODUCTION

**FIGURE 1:**
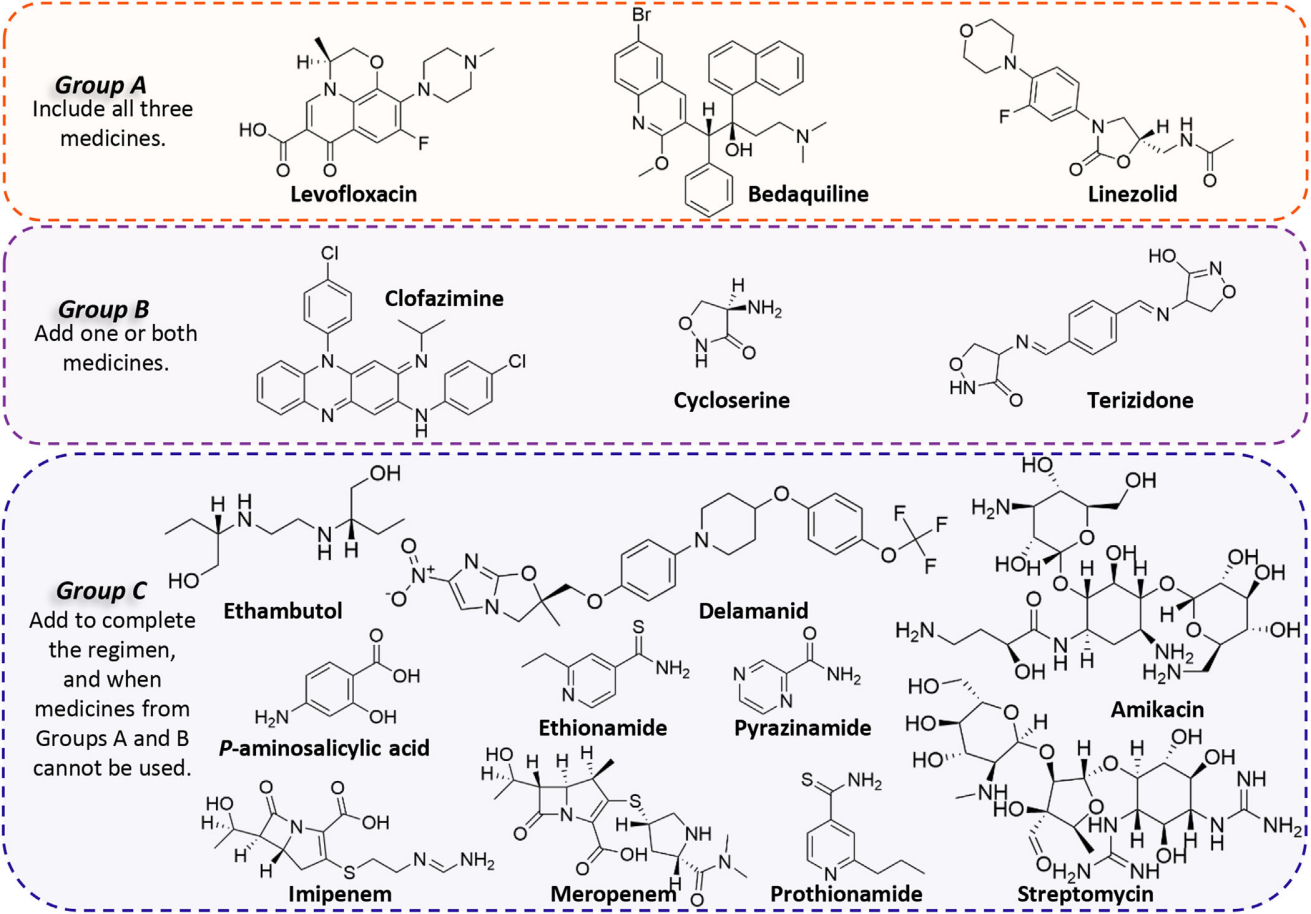
Grouping of medicines recommended for use in longer multidrug‐resistant TB (MDR‐TB) regimens by the WHO.^2^ Group A drugs are strongly recommended for inclusion in all longer MDR‐TB regimens containing fluoroquinolones, bedaquiline, and linezolid. Group B drugs were recommended to add one or two to the regimen to improve outcomes. Group C drugs were recommended as a secondary option to Group A and B drugs.

Tuberculosis (TB), caused by *Mycobacterium tuberculosis* (*Mtb*), remains a significant global health problem, despite advances in diagnosis and treatment. Approximately 10 million new cases of TB are diagnosed each year, with over a million deaths attributed to the disease.^1^ TB is particularly prevalent in developing countries, where coinfection with human immunodeficiency virus (HIV) and poor living conditions are common.^2^ Additionally, the emergence of drug‐resistant strains of *Mtb* poses a significant challenge to global TB control efforts.^3^ The current standard regimen for drug‐sensitive TB involves a combination of drugs, including isoniazid (INH), rifampicin (RIF), pyrazinamide (PZA), and ethambutol (EMB). However, treatment can be lengthy and costly, with patients often requiring 6 months of therapy.^4^ Moreover, the emergence of multidrug‐resistant TB (MDR‐TB) and extensively drug‐resistant TB (XDR‐TB) has further complicated the treatment landscape (Figure 1).

**FIGURE 2:**
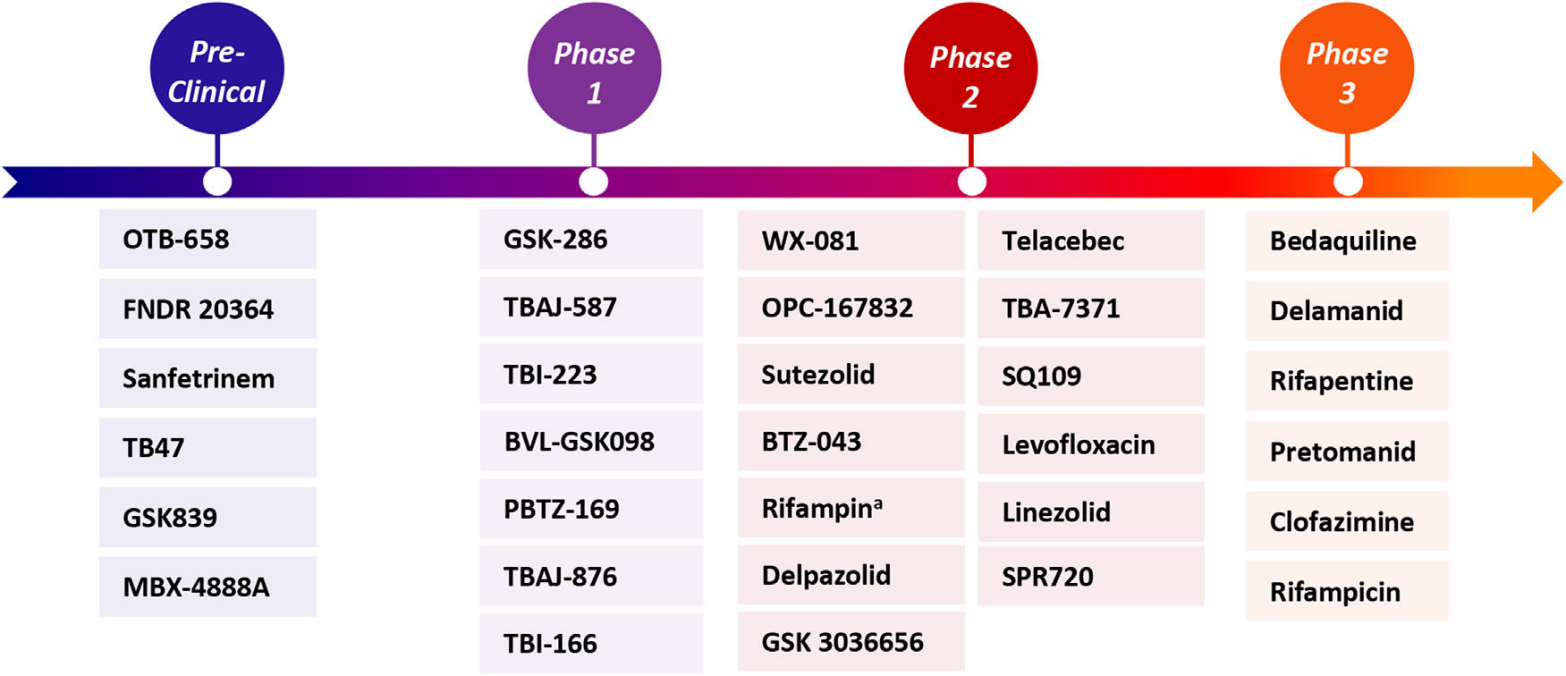
Current global clinical pipeline of new tuberculosis drugs based on information provided by the Working Group for New TB Drugs (WGND).^156^ ^a^Trial of high‐dose Rifampin in patients with TB.

While progress has been made in the development of new TB drugs, there remains a significant need for novel treatment options. Several drug candidates are presently in diverse phases of clinical development (Figure 2), comprising reoptimized adaptations of current TB drugs and some involving original mechanisms of action. Additionally, several new targets for drug development have been identified.^4^ In the context of the coronavirus disease 2019 (COVID‐19) pandemic, the impact of TB on global health has become even more severe. Individuals who have recovered from COVID‐19 have been found to have a higher risk of developing TB, likely due to the negative impact of COVID‐19 on the immune system.^5^ Therefore, new TB treatment drugs remain an urgent research priority.

This review aims to identify potential targets that show promise for the development of new therapeutic approaches to treat TB, based on an understanding of the pathogenesis of the disease. The ability of *Mtb* to survive in the microenvironment of the human host is one of the greatest challenges faced when developing new drugs. We first elucidate the pathogenesis of *Mtb*, which encompasses the phases of invasion, proliferation, latency, and revival of the pathogen. By understanding the mechanisms, we can identify new drug targets and advance the development of more effective TB treatments. In the pathogenic process, *Mtb* acts as a pathogen causing TB, so targets within the bacterium and drugs that directly kill *Mtb* are of great interest. We reviewed all kinds of targets and drugs targeting *Mtb*. Simultaneously, *Mtb* provokes an immunoreaction in its host, while skillfully tampering with vital cellular processes, leading to protracted treatment and unfavorable prognosis. This review will summarize the latest research identifying high‐value targets in host‐targeted therapies and describe the related modulaters.

## 2 PATHOGENESIS OF *Mtb*

**FIGURE 3:**
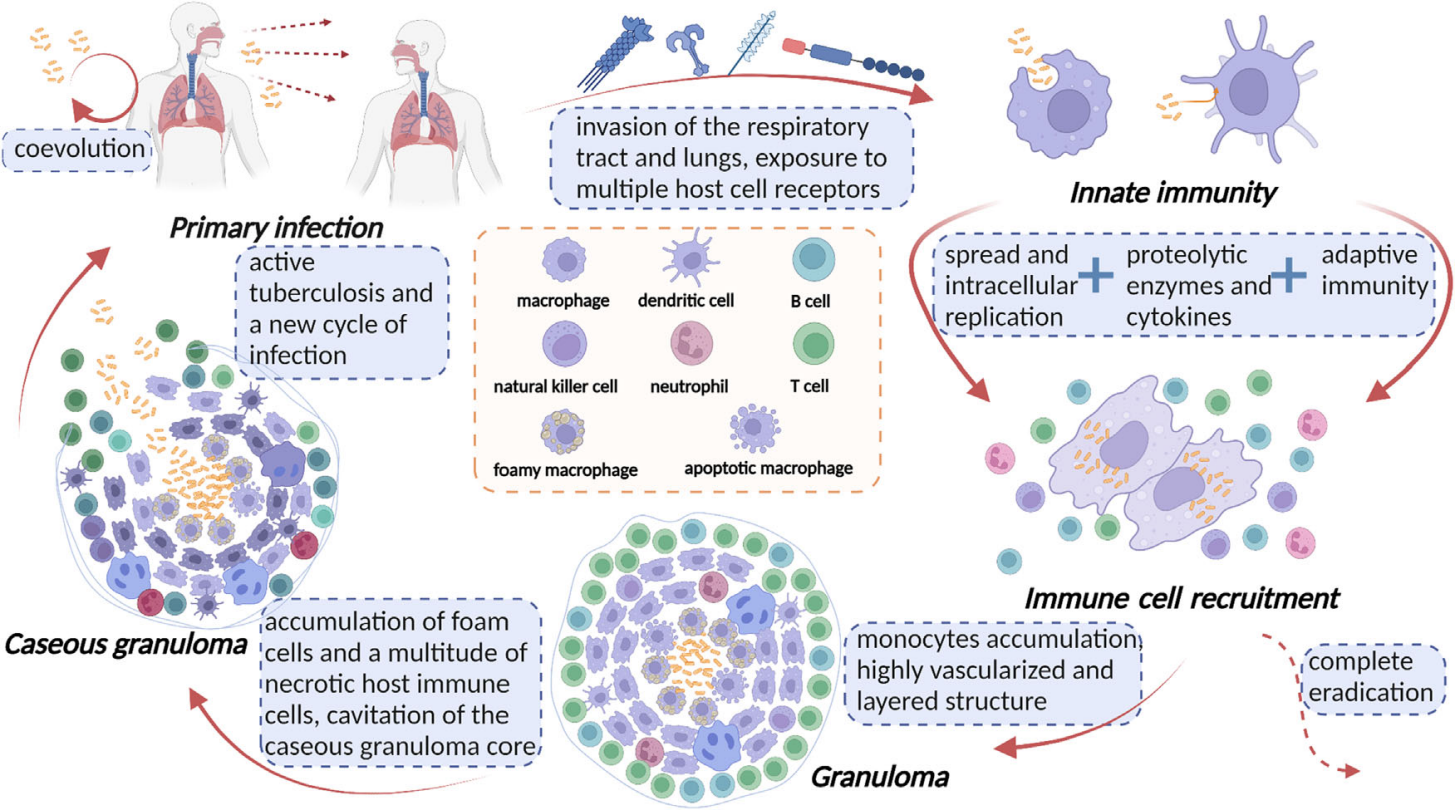
Pathophysiology of pulmonary TB. Upon entering the respiratory tract and lungs of the host, *Mtb* incites an innate immune response and is engulfed by pivotal immune cells such as macrophages and dendritic cells. Subsequently, *Mtb* replicates within these cells as more immune cells are recruited to the site of infection. Whilst it is possible for the host to completely eliminate *Mtb* at this stage, the formation of solid granulomas is often prompted. These granulomas are composed of foam cells derived from macrophages, as well as a multitude of necrotic immune cells, culminating in caseous granulomas that eventually rupture and release bacteria, giving way to the subsequent development of active TB. Ultimately, these *Mtb* bacteria are released as infectious aerosol droplets, reinstating a new cycle of infection. The elements in the figure were drawn using BioRender online tool (https://biorender.com).

*Mtb* complex (MTBC) has persistently accompanied our species, anatomically modern humans, during our evolutionary journey and widespread dispersion across the globe throughout the past 70 millennia. *Mtb* initiates its life cycle upon invasion of the respiratory tract and lungs (Figure 3), as the microorganism is classically believed to thrive only within living organisms.^6^, ^7^ This initial interaction, commonly referred to as primary infection, marks the onset of contact between the pathogen and the respiratory system. There has been a contention that the coevolution between *Mtb* and its human host does not follow the conventional trajectory of an evolutionary arms‐race. Instead, it is suggested to be characterized by mechanisms of manipulation.^7^ Following exposure to multiple host cell receptors (including toll‐like receptors [TLRs], C‐type lectin receptors [CLRs], dendritic cells [DCs], mannose receptors [MRs], and NOD‐like receptors [NLRs]) and internalization by alveolar macrophages and DCs, *Mtb* undergoes a phase of intracellular replication. Subsequently, infected cells spread to lymph nodes while attempting to destroy the bacteria with various proteolytic enzymes and cytokines (e.g., tumor necrosis factor alpha [TNF‐α] and interferon gamma [IFN‐γ]), perpetuating spread of the pathogen throughout the host's lung parenchyma upon arrival.^8^ Activation of macrophages results in recruitment of additional innate immune cells, thereby fostering inflammatory responses that contribute to host defense against the pathogen. Neutrophils demonstrate a heightened intensity of phagocytosis compared to macrophages, coupled with elevated levels of reactive oxygen species (ROS)‐mediated oxidative burst.^9^ Upon recruitment of lymphocytes to the site of infection, a cascade of cell‐mediated immune responses is initiated, leading to the arrival of additional immune cells aimed at localizing bacterial colonization and inhibiting their proliferation.^10^, ^11^ During the initial stages, a characteristic delay is observed in the T‐cell response, which aids the pathogen in establishing a persistent infection.^10^ At this point, complete eradication of *Mtb* is feasible if the host immune capacity remains intact.^12^ In most cases, however, the immune response is not sufficient to eradicate *Mtb*. Monocytes accumulate in the vicinity of infected macrophages, precipitating the formation of solid granulomas, a pathological feature characteristic of TB. Additionally, *Mtb* can traverse the mucosa upon infection of alveolar epithelial cells and stimulation of cellular necrosis, further exacerbating pathogenesis.^13^, ^14^ Hence, the gathered data suggest that local immune responses play a pivotal role in mitigating progression of TB disease.^15^

Despite being formed with the intent of containing the spread of bacteria, granulomas can also serve as a refuge for bacterial populations, thus evading further recognition and removal by the host immune system, resulting in a clinically defined state of latent TB infection (LTBI).^12^, ^16^ Ensuring screening, diagnosis, and treatment of LTBI is critical in facilitating the global decline in TB incidence and ultimately achieving TB elimination. During early development, the granuloma is highly vascularized (via vascular endothelial growth factor (VEGF)) and the vessels have extensive lymphocytic cuffs. As the granuloma progresses, macrophages undergo differentiation into various morphotypes, resulting in the formation of a layered structure with a layer of lymphocytes aggregated outside a fibrous cuff surrounding a macrophage‐rich layer. In this scenario, patients harbouring granulomas are asymptomatic and noninfectious.^17^, ^18^ Studies have revealed that *Mtb* harnesses the production of mycolic acid (MA) to instigate differentiation of macrophages into foam cells. The core of the granuloma can give rise to a caseous granuloma, characterised by the accumulation of foam cells and a multitude of necrotic host immune cells.^19^ During late‐stage TB, cavitation of the caseous granuloma core may ensue, resulting in the release of bacteria and consequent progression to active TB disease.^20^ Consequently, the reactivation of LTBI, and ensuing progression to symptomatic disease, can enable transmission of the bacteria to a new host, perpetuating a new cycle of infection. Moreover, *Mtb* can disseminate through the bloodstream^21^ and lymphatic endothelial cells,^22^ disseminating beyond infected lungs and leading to the development of extrapulmonary TB (EPTB). EPTB can manifest in nearly any part of the body, akin to lymph nodes, pleura, genitourinary system, bones and joints, and other organs.^23^

Pursuing an in‐depth comprehension of the pathogenesis of TB may unlock novel therapeutic avenues. Despite having been identified and isolated over a century ago, *Mtb* has continued to inflict protracted distress and fatalities worldwide. Moreover, the latest data from the World Health Organization (WHO) regarding drug‐resistant TB are alarming, with approximately 450,000 new cases of RIF‐resistant TB reported in 2021.^1^ The emergence of drug‐resistant strains of TB correlates with the epidemic of HIV, and early incidence of drug‐resistant TB epidemics were witnessed primarily among HIV–*Mtb* coinfected patients.^24^, ^25^ Furthermore, the detrimental effects of the COVID‐19 pandemic have impeded the identification and management of TB cases, undermining the gains made in combatting TB in recent years.^5^, ^26^ Therefore, it is imperative to recognize the urgency of discovering effective therapies for TB.

Several studies have explored the potential use of Bacillus Calmette‐Guérin (BCG) as prophylaxis for TB; however, results indicate that BCG exerts suboptimal effects on immune memory.^27^, ^28^, ^29^ Nonetheless, promising breakthroughs in the analysis of pathogen–host interactions and evolutionary investigations of *Mtb* offer prospective avenues for identifying pathogenicity and virulence factors that could catalyze the development of novel therapies for TB.^30^

## 3 DRUG TARGETS AND INHIBITORS TARGETING *Mtb*

Invasion of *Mtb* causes TB and its ability to replicate and maintain using host cellular mechanisms makes it a major target for killing by drugs. The publication of the whole genome sequencing of *Mtb* has advanced our understanding of the molecular biology of this bacterium, making it easier to identify specific targets. In fact, the most potent drugs targeting *Mtb* primarily aim at eradicating the pathogen currently.^31^ Unquestionably, novel drug discovery and development efforts concentrate predominantly on direct *Mtb* killing. This section reviews the drug targets and inhibitors targeting *Mtb*, which are classified according to the different actions associated with bacterial survival and are discussed in different subsections.

### 3.1 Cell wall synthesis and assembly

*Mtb*, the causative agent of TB, possesses an atypical cellular envelope comprising primarily lipids and carbohydrates. This envelope is characterized by the complex mycolyl‐arabinogalactan‐peptidoglycan (mAGP) and phosphatidyl‐myo‐inositol‐based lipoglycans. The mAGP complex is constructed from several critical components, including peptidoglycan (PG), arabinogalactan (AG), MA units, and the indispensable lipoarabinomannan (LAM).^32^, ^33^ It is estimated that the cellular envelope synthesis and assembly pathway of *Mtb* harbors a minimum of 60 prospective enzymatic targets.^4^, ^34^, ^35^ The targets described in this section involve the synthesis of MA, AG, LAM, PG, and the transport of MA.

#### 3.1.1 MA biosynthesis

MA, a crucial component of *Mtb*’s cellular envelope, is synthesized predominantly through the fatty acid synthesis (FAS) pathway. It is noteworthy that unlike mammalian FAS‐I synthase, which utilizes a multidomain protein, FAS in *Mtb* is carried out by a combination of FAS‐I synthase and several FAS‐II monofunctional enzymes. The FAS‐II type system is responsible for the extension of acyl‐CoA (C16:0 to C18:0), the products of de novo synthesis by FAS‐I.^36^ This unique contrast renders the FAS pathway a viable therapeutic target for pharmacological agents aimed at combating *Mtb* infection.

##### FAS‐II pathway

InhA, an enoyl‐acyl carrier protein (ACP) reductase, facilitates the reduction of long‐chain *trans*‐2‐enoyl‐ACP by forming covalent adducts between nicotinamide cofactors and enoyl‐CoA substrates in the FAS‐II pathway.^37^ The role of several first‐line anti‐TB drugs such as INH, ethionamide (ETH, Group C), and prothionamide (PTH, Group C)^38^, ^39^ (Table 1) demonstrates that InhA is an important therapeutic target for the treatment of *Mtb* infection.

**TABLE 1:** Biological targets and inhibitors targeting *Mtb*.

Mechanism of action		Target	Typical compound	Drug stage for TB
Cell wall synthesis and sssembly	MA^a^ biosynthesis	Enoyl‐acyl carrier protein reductase (InhA)	Isoniazid^38^	Approved
			Ethionamide^39^	Approved
		β‐Ketoacyl synthase (KasA)	Thiolactomycin^66^, ^67^, ^68^, ^69^	Biological test
		Fatty acid degradation protein D32 (FadD32)	Quinoline‐2‐carboxamide^78^	Biological test
		Polyketide synthase 13 (Pks13)	TAM16^79^	Biological test
		Mycolic acid methyltransferase 4 (MmaA4)	SADAE^88^	Biological test
		Cyclopropane mycolic acid synthase (CMAS)	/	In silico docking
	AG biosynthesis	*N*‐acetylglucosamine‐1‐phosphate transferase (WecA)	CPZEN‐45^97^	Preclinical
	LAM biosynthesis	Arabinosyl transferase C (EmbC)	Amikacin^106^	Approved
		Decaprenylphosphoryl‐β‐D‐ribose‐2′‐epimerase (DprE1)	PBTZ‐169^110^	Phase I
			OPC‐167832^111^	Phase II
			TBA‐7371^112^	Phase II
			BTZ‐043^113^	Phase II
	PG biosynthesis	Alanine racemase (Alr)	Cycloserine^130^, ^131^	Approved
		D‐alanyl‐D‐alanine ligase (Ddl)		
		L,D‐transpeptidase type 2 (Ldt_Mt2_)	Meropenem^138^	Approved
		Phospho‐*N*‐acetylmuramoyl‐pentapeptide transferase (MurX)	SQ641^148^, ^149^	Preclinical
	MA transporter	Mamalian membrane protein large 3 (MmpL3)	SQ109^155^	Phase II
Lipid metabolism		Aspartate decarboxylase (PanD)	Pyrazinamide^171^, ^172^	Approved
Protein synthesis and breakdown		Ribosome	TBI‐223^191^	Phase I
		Leucyl‐tRNA synthase (LeuRS)	GSK656^194^	Phase II
		Caseinolytic protease P (ClpP)	EZ120^200^	Preclinical
		Proteasome	Phenylimidazole^210^	Preclinical
		Proteasome accessory factor A (PafA)	ST1926^213^	Biological test
Amino acid synthesis and metabolism		Ser/Thr protein kinases (protein kinase G, PknG)	AX20017^220^	Biological test
			NU‐6027^224^	Biological test
		Shikimate pathway (3‐dehydroquinate synthase, DHQS)	IMB‐T130^228^	Biological test
		Tryptophan synthase (TrpAB)	BRD4592^233^	Preclinical
Nucleotide synthesis		Type I topoisomerase (topoI)	m‐AMSA^241^	Biological test
		Type II topoisomerase (DNA gyrase)	Levofloxacin^244^	Phase II
			SPR720^246^	Phase II
		RNA polymerase (RNAP)	Rifampicin^252^	Approved
		transcriptional repressor of *ethA* (EthR)	BVL‐GSK098^257^, ^258^	Phase 1
Energy metabolism		Type II NADH dehydrogenase (NDH‐2)	Clofazimine^263^	Phase III
			TBI‐166^264^	Phase I
		β Subunit of cytochrome *bc*_1_ complex (QcrB)	Telacebec^268^	Phase II
		ATP synthase	Bedaquiline^276^	Phase III
			TBAJ‐876^280^	Phase I
			TBAJ‐587^280^	Phase I
			WX‐081^281^	Phase II
Virulence	Two‐component system	PhoPR	Ethoxzolamide^294^	Biological test
			Artemisinin^299^	Biological test
		DosRST	HC102A^299^	Biological test
			HC103A^299^	Biological test
	ESX‐1 secretion system	ESAT‐6 secretion system‐1 (ESX‐1)	BBH7^306^	Biological test
			BTP15^306^	Biological test
Folic acid and mycobactin biosynthesis		Dihydrofolate reductase (DHFR)	*P*‐aminosalicylic acid^308^	Approved
			AF‐353^311^	Biological test
Metal uptake		Iron‐dependent regulator (IdeR)	/	In silico docking
Cholesterol metabolism		Adenylyl cyclase (AC)	GSK2556286^316^	Phase I

^a^ MA, mycolic acid; AG, arabinogalactan; LAM, lipoarabinomannan; PG, peptidoglycan.

Various studies have demonstrated that INH, ETH, and PTH exert their inhibitory effect on InhA by forming an INH/ETH/PTH‐NAD+ adduct, requiring prior activation.^40^, ^41^, ^42^, ^43^ *Mtb* possesses two enzymes—KatG, a catalase‐peroxidase,^44^ and EthA, a monooxygenase^45^—which catalyze the generation of free radical inhibitors that bind to NADH to form binary complexes.^38^ Recent research has revealed that during the catalytic cycle of InhA,^37^ NADH cofactors and octenoyl‐CoA substrates form covalent adducts, including a C2‐ene adduct, which offers insight into the function of key amino acid residues at the active site of InhA.

The emergence of drug resistance due to mutations of activating enzymes is a formidable challenge in the case of INH, ETH, and PTH. As a result, researchers have shifted their focus to identifying compounds that can directly bind to InhA, spurred by the discovery of targets for the broad‐spectrum fungicide triclosan.^46^, ^47^, ^48^ Promising direct inhibitors of InhA have been identified, such as diphenyl ethers,^47^, ^49^, ^50^ pyrrolidine carboxamides,^51^ arylamides,^52^ indole‐5‐Amides,^53^ pyridones,^54^ imidazopiperidines,^55^ thiadiazoles,^56^ diazaborines,^57^ and benzimidazoles.^58^ Most InhA inhibitors occupy the substrate binding sites. However, pyridomycin,^59^ produced by *Streptomyces pyridomyceticus* ^60^, ^61^ or *Dactylosporangium fulvum*,^62^ represented the first direct competitive inhibitor of NADH binding with specific activity against mycobacteria. The crystal structures of InhA, either WT (PDB: 4BII) or the INH‐resistant S94A mutant (PDB: 4BGE),^63^ bound to pyridomycin were determined and confirmed that pyridomycin occupied simultaneously the NADH and the substrate binding sites.^64^

Similar to InhA, β‐ketoacyl synthase KasA also plays a pivotal role in the elongation of long‐chain fatty acids by facilitating the initial step of the FAS‐II pathway.^65^ Thiolactomycin is a thiolactone natural product that inhibits all three annotated *Mtb* β‐ketoacyl synthases, including KasA, in various functional assays.^66^, ^67^, ^68^, ^69^ DG167, an indazole sulfonamide, was first identified as an antitubercular through a high‐throughput screening (HTS) campaign.^70^, ^71^ The X‐ray crystallography has been employed to determine the molecular structure of this compound while it was bound to KasA.^72^, ^73^ The in vivo efficacy of transposed indazole sulfonamide derivatives based on DG167 has shown substantial improvement in an acute infection model of *Mtb* in mice.^74^

##### Fatty acid degradation protein D32/Polyketide synthase 13 crosstalk

Fatty acid degradation protein D32 (FadD32) acts as a fatty acyl‐AMP ligase, transferring the resulting acyl‐adenylate to specific thioester acceptors.^75^ Polyketide synthase 13 (Pks13) is a module encoding several enzymatic and transport functions to the viability and virulence of *Mtb*.^76^ Once produced by the FadD32 enzyme, the resulting acyl‐AMPs are specifically transferred to the ketosynthase domain of Pks13 after binding to the phosphopantetheinyl moiety of its N‐terminal ACP domain (N‐ACP(Pks13)). Together, FadD32 and N‐ACP(Pks13) constitute the initiation module of the mycolic condensation system.^77^ Recent research reports that several quinoline‐2‐carboxamides effectively reduce the mycobacterial burden in mouse lungs by inhibiting FadD32 activity upon oral administration.^78^ TAM16, a benzofuran derivative, has also been identified as a promising inhibitor of Pks13.^79^ Additionally, coumarin^80^ and chromen‐4‐one derivatives^81^ have likewise demonstrated Pks13 inhibition, although a continued focus on optimizing in vivo therapeutic efficacy is warranted.

Delamanid (DLM, Group C) and pretomanid (Pa) are prodrugs that need to be activated by the deazaflavin F420‐dependent nitroreductase enzyme.^82^, ^83^ DLM and Pa, two promising nitroimidazole candidates, exert their anti‐*Mtb* effects by selectively inhibiting the biosynthesis of specific MA, such as methoxy‐ and keto‐MA.^84^ By contrast, INH inhibits the synthesis of all MA classes—methoxy‐, keto‐, and α‐MA.^85^ The precise enzyme targets of nitroimidazoles in cell wall biosynthesis have not yet been identified, though current research supports their multitargeted nature. Further, Pa has been shown to act as a direct nitric oxide (NO) donor, offering insight into its mechanisms for *Mtb* killing under hypoxic, nonreplicating conditions.^86^ This is an encouraging prospect for the treatment of on LTBI. Spontaneous drug‐resistant mutants of *Mtb* were found to carry mutations in MA methyltransferases, MmaA4 and MmaA2.^87^ Crystallographic studies identify the interaction of *S*‐adenosyl‐*N*‐decyl‐aminoethyl (SADAE) with MmaA4 and provide ideas for inhibitor design.^88^ MAs cyclopropanation contributes to virulence, antibiotic resistance, and intracellular survival and is catalyzed by enzymes of the cyclopropane MA synthase (CMAS) family.^89^, ^90^ MA cyclopropane synthase PcaA (also known as UmaA2), which is essential for the nucleation morphology of *Mtb* ^91^ and is expressed at high levels during *Mtb* dormancy,^92^ appears to be a potential target for dormant mycobacteria. In addition to PcaA, the cyclopropane synthases CmaA1 and CmaA2 are also involved in the cyclopropanation of MAs.^93^, ^94^ Several skeletons have been obtained by in silico docking,^92^, ^95^, ^96^ and subsequent biochemical validation and optimization are expected. These targets have received limited research attention and hold promising potential for further investigation and development.

#### 3.1.2 AG biosynthesis

##### *N*‐acetylglucosamine‐1‐phosphate transferase

*N*‐acetylglucosamine‐1‐phosphate transferase (WecA) responsible for initiating AG biosynthesis in *Mtb*, has been identified as a potential target for the caprazamycin derivative CPZEN‐45.^97^ This preclinical drug candidate has shown promise as an inhalation treatment option for TB.^98^, ^99^ Studies have demonstrated that transcriptional silencing of the gene encoding WecA has a bactericidal effect on *Mtb* both in vitro and in vivo.^100^ The discovery of novel WecA inhibitors has been supported by medium‐ to HTS methods targeting WecA. Such screening methods have contributed to the identification of potential inhibitors of WecA, which can serve as lead compounds for the development of new drugs to treat TB.^101^

#### 3.1.3 LAM biosynthesis

##### Arabinosyl transferase C

Arabinosyl transferases EmbA, EmbB, and EmbC are critical components of the mycobacterial cell wall biosynthesis pathway. While EmbA and EmbB are known to interact and form a heterodimeric complex, EmbC functions as a homodimeric enzyme.^102^ Specifically, EmbA and EmbB are involved in the formation of the terminal hexaarabinofuranoside motif in AG,^103^ while EmbC is responsible for chain lengthening of LAM.^104^ Recent data from crystallography and overexpression studies suggest that EMB (Group C) competes with substrates for binding to the EmbB and EmbC subunits.^102^, ^105^ Notably, a high‐throughput virtual screening of the United States Food and Drug Administration (US FDA) library has identified two additional EmbC inhibitors, terlipressin and amikacin^106^ (Group C).

##### Decaprenylphosphoryl‐β‐d‐ribose‐2′‐epimerase

Decaprenylphosphoryl‐β‐D‐ribose‐2′‐epimerase (DprE) is a heterodimeric diastereoselective enzyme containing DprE1 and DprE2.^107^ DprE1 catalyzes the two‐step epimerization of decaprenyl‐phospho‐ribose to decaprenyl‐phospho‐arabinose, the precursor for AG and LAM synthesis.^108^ DprE1 was originally discovered as a major target of benzothiazinones, which have demonstrated potent antimicrobial activity against *Mtb*.^109^ Currently, several DprE1 inhibitors are in various stages of clinical development, as of February 2023. These include PBTZ‐169^110^ (macozinone, phase I), OPC‐167832^111^ (phase II), TBA‐7371^112^ (phase II), and BTZ‐043^113^ (phase II). Remarkably, both BTZ‐043 and PBTZ‐169 are covalent inhibitors of DprE1. The nitro group of the benzothiazinone scaffold is reduced to form its nitroso derivative, which binds to the Cys387 residue in DprE1, resulting in irreversible enzyme inhibition.^114^, ^115^ In contrast, OPC‐167832 is a carbostyril derivative and TBA‐7371 is a 1,4‐azaindole, both of which are noncovalent DprE1 inhibitors. Recent research have identified a variety of new DprE1 inhibitors, including benzothiazinones containing 2‐benzyl‐2,7‐diazaspiro[3.5]nonane,^116^ benzothiopyranones,^117^ morpholino‐pyrimidines,^118^ hydantoins,^119^ thiophene‐arylamides,^120^ 4‐aminoquinolone piperidine amides,^121^ 2‐carboxyquinoxalines,^122^ *N*‐alkyl‐5‐hydroxypyrimidinone carboxamides,^123^ selamectin,^124^ with promising antimycobacterial activity.^125^

#### 3.1.4 PG biosynthesis

##### d‐Alanyl‐d‐alanine dipeptide synthesis pathway

Alanine racemase (Alr) is a crucial pyridoxal 5′‐phosphate‐dependent amino acid racemase enzyme that facilitates the conversion of l‐alanine to d‐alanine, which is utilized by bacterial cell walls for PG biosynthesis.^126^ d‐Alanyl‐d‐alanine ligase (Ddl) is a multistructural domain protein that is dependent on adenosine triphosphate (ATP) and is involved in the biosynthesis of PG precursors. Ddl catalyzes the ligation of two d‐alanine molecules into one d‐alanyl‐d‐alanine dipeptide.^127^ The inhibition of both Alr and Ddl enzymes in *Mtb* can lead to a significant weakening of the cell wall, making these enzymes crucial targets for intervention.^128^ Terizidone (Group B), a compound comprising of two cycloserine (CS) moieties linked to a terephthalaldehyde molecule, undergoes in vivo hydrolysis to CS and exhibits activity against these enzymes.^129^ CS (Group B), a second‐line medication utilized in the management of TB and MDR‐TB, is effective in inhibiting the synthesis of PG by concurrently targeting the Alr and Ddl enzymes.^130^, ^131^ However, CS has limited clinical utility owing to its nonspecific nature. Other Alr inhibitors, including alanine phosphonates and thiadiazolidinones that are currently underutilized in clinical practice, similarly lack specificity due to their effects on multiple other phosphate‐dependent enzymes.^126^ Although several Ddl inhibitors have been demonstrated to be effective at the molecular and cellular level, they require further development for clinical application.^127^, ^132^

##### l,d‐Transpeptidase type 2

The presence of β‐lactamase, BlaC, in *Mtb* has long impeded the development of β‐lactam anti‐TB antibiotics.^133^ However, the combination of carbapenem antibiotics and β‐lactamase inhibitors has recently been revisited as a strategy in the fight against TB. Studies have shown that meropenem‐clavulanate (Group C) is highly effective against extensively XDR‐TB.^134^, ^135^ Ldt_Mt2_ is a critical enzyme involved in cell wall synthesis, virulence and amoxicillin tolerance of *Mtb*.^136^, ^137^ Complexation of l,d‐transpeptidase type 2 (Ldt_Mt2_) and meropenem demonstrates that inactivation of Ldt_Mt2_ may be the main mechanism of meropenem‐clavulanate effectiveness against *Mtb*.^138^ Hybrid quantum mechanics/molecular mechanics offer a potential avenue to obtain active molecules that inhibit Ldt_Mt2_^139^. Biapenem, a carbapenem that boasts superior stability, has been evaluated against RIF‐resistant *Mtb*.^140^, ^141^ Recent investigations have implicated both reversible reactions and nonhydrolytic off‐loading reactions from the cysteine transpeptidase Ldt_Mt2_ in the effectiveness of meropenem.^142^ These findings provide a direction for future optimization of next‐generation anti‐TB carbapenems. The development of carbapenem antibiotics is a remarkable accomplishment, and it is essential to proceed quickly with in vitro and in vivo experimental validations to establish effective treatment regimens for XDR‐TB. Recently, a low‐molecular‐weight organoselenium compound ebselen was shown to inhibit Ldt_Mt2_, suggesting that cysteine‐reactive reagents may act as potential Ldt_Mt2_ inhibitors.^143^

##### Phospho‐*N‐*acetylmuramoyl‐pentapeptide transferase

Phospho‐*N‐*acetylmuramoyl‐pentapeptide transferase (MurX), also called translocase I, converts UDP‐MurNAc‐pentapeptide into prenyl‐MurNAc‐pentapeptide (lipid I) in PG biosynthesis.^144^ MurX has been identified as the target for five families of nucleoside antibacterial natural products, which include the tunicamycins, mureidomycins, liposidomycins, muraymycins, capuramycins, and sansanmycins.^145^, ^146^, ^147^ The capuramycin analogue SQ641 has been shown to be effective in killing *Mtb* by inhibiting MurX.^148^, ^149^ However, to accommodate higher drug loads, SQ641 requires a phospholipid‐based nanoemulsion formulation.^150^ This formulation allows for increased drug delivery and improved efficacy in treating TB. The chemical properties of lipid I renders MurX enzyme assays impractical for screening and lacks reproducibility of the enzyme assays.^151^ However, in vitro experiments have shown that a water‐soluble analogue of lipid I can be used as a substitute to quantify the inhibitory activity of library molecules against MurX.^152^ This approach has allowed researchers to identify potential new inhibitors of MurX that could be further developed for use in TB treatment.

#### 3.1.5 MA transporter

##### Mamalian membrane protein large 3

Trehalose monomycolates (TMMs), crucial components in the robust barrier against *Mtb*, are transferred from the cytoplasm to the periplasm via the Mamalian membrane protein large 3 (MmpL3). Further, TMMs act as the essential building blocks for the synthesis of MAs, an indispensable feature of the protective envelope of *Mtb*, rendering the latter vulnerable to the effects of TMM depletion.^153^ The discovery of knockdown mutants has illuminated the critical role of MmpL3 in both the replication and activity of *Mtb*, thereby enhancing our comprehension of its multifaceted pathogenesis.^154^ Notably, recent advances have led to the discovery of several structurally unique MmpL3 inhibitors, including the 1,2‐ ethylenediamine SQ109, which has successfully advanced to clinical Phase 2 trials.^155^, ^156^ Other inhibitors, such as the adamantyl urea, the 1,5‐diarylpyrrole, the tetrahydropyrazolopyrimidine, and the indolecarboxamide, have demonstrated significant potential and warrant further investigation.^157^, ^158^, ^159^, ^160^

### 3.2 Lipid metabolism

Complex lipids of *Mtb* demonstrate a striking ability to function as critical effector molecules that engage in dynamic interactions with the host. By modulating host metabolism and inciting a robust immune response, these lipids play a paramount role in shaping both the mycobacterium's own physiology and that of the host cells. This section reviews the discovery process of targeting aspartate decarboxylase (PanD).

#### 3.2.1 Aspartate decarboxylase

PanD is a key enzyme implicated in the biosynthesis of β‐alanine, a critical precursor molecule for pantothenate and CoA biosynthesis, derived from l‐asparate.^161^ Pantothenate, also known as vitamin B5, serves as a crucial building block for CoA production, which is primarily involved in the synthesis of mycolic MA.^162^ Notably, PanD is conspicuously absent in mammalian systems, thereby indicating the therapeutic potential of PanD inhibitors.^163^ PanD, along with pantothenate synthase, has piqued the interest of researchers in the development of PZA, a Group C anti‐TB drug.^164^ PZA functions as a prodrug that is enzymatically activated by nicotinamidase or pyrazinamidase (PZAse), encoded by the *pncA* gene in *Mtb*, thereby promoting its conversion into the pharmacologically active pyrazinoic acid (POA).^165^ PZA's discovery was the result of a therapeutic test on *Mtb*‐infected mice.^166^ Although PZA exhibited limited anti‐TB activity in vitro in the standard neutral pH broth medium, the exact mechanism remained an enigma.^167^, ^168^ Nevertheless, years of research have resulted in significant breakthroughs in understanding PZA's mode of action. Genome sequencing data have identified mutations in the *panD* gene responsible for PanD production in PZA‐resistant strains, independent of mutations in *pncA* and *rpsA*, which were previously presumed as targets for PZA.^169^, ^170^ Here, it was discovered that while PZA was ineffective, the active metabolite POA competes with PanD, thereby disrupting the CoA biosynthetic pathway.^171^, ^172^ This novel insight may help clarify why POA can effectively target *Mtb* under nonreplicating conditions, leading to a reduction of the treatment regimen by three months.^169^

The other enzymes of the pantothenate synthetase pathway, ketopantoate hydroxymethyltransferase PanB, pantothenate synthetase PanC, and ketopantoate reductase PanE, are also worthy targets for development.^161^, ^173^, ^174^ Research on pantothenate synthetase inhibition is currently focused on two main approaches. The first approach involves the synthesis of nonreactive analogues of the reaction intermediate.^175^, ^176^ The second approach involves identifying hits for pantothenate synthetase inhibition through HTS, followed by structure‐based validation to determine their efficacy and safety as potential drug candidates.^177^ These methods contribute pantoyl adenylate analogues,^175^ nafronyl oxalate,^178^ actinomycin D,^179^ 3‐phenyl‐4,5,6,7‐tetrahydro‐1H‐pyrazolo[4,3‐c]pyridine derivatives,^180^ and 5‐tert‐butyl‐*N‐*pyrazol‐4‐yl‐4,5,6,7‐tetrahydrobenzo[d]isoxazole‐3‐carboxamide derivatives^181^ as pantothenate synthetase inhibitors. In addition to the two approaches previously mentioned, there are also groups focused on fragment growing,^177^ virtual screening,^182^ and molecular hybridization^183^, ^184^ aimed at providing compounds with higher whole‐cell activity. These approaches can lead to the development of novel compounds with improved efficacy and safety for the treatment of TB.

### 3.3 Protein synthesis and breakdown

Protein synthesis represents an indispensable process vital for the survival and replication of all living organisms, taking place within the ribosomes of cells.^185^ This section describes the *Mtb* ribosome and the leucyl‐tRNA synthase (LeuRS) involved in protein synthesis, as well as the caseinolytic protease P (ClpP) system and the proteasome system responsible for the degradation of intracellular damaged proteins.

#### 3.3.1 Ribosome

In the instance of *Mtb*, the ribosomal machinery forms a large, functional 70S ribosome consisting of a 2.7 MDa complex.^186^ Notably, the 50S large subunit contains 37 ribosomal proteins, as well as 23S and 5S rRNAs, whereas the smaller 30S subunit is comprised of 21 ribosomal proteins and 16S rRNA.^187^, ^188^ In particular, Mycobacterial‐specific protein Y successfully binds to the 30S subunit, inducing ribosomal hibernation and mediating resistance to aminoglycoside antibiotics.^189^ This phenomenon is implicated in the development of nonreplicating *Mtb*,^190^ highlighting the potential of ribosomal targeting to impede protein synthesis. Aminoglycosides, oxazolidinones (including TBI‐223,^191^ currently in phase I, sutezolid,^192^ and delpazolid,^193^ presently in phase II) represent classes of drugs effective for targeting ribosomes.

#### 3.3.2 Leucyl‐tRNA synthase

LeuRS, belonging to the class I aminoacyl‐tRNA synthase subgroup, represents a critical player in intracellular transport. Specifically, GSK656 (phase II), a benzoxazole compound, has shown considerable potential as a specific inhibitor of LeuRS through its targeting of the catalytic site of hydrolysis of incorrectly ligated aminoacylated tRNA.^194^ Unlike the initial lead compounds, GSK656 exhibits enhanced selectivity over human cytoplasmic LeuRS while effectively inhibiting protein synthesis in intact human cells.^195^ Upon computer screening, *N‐*benzylidene‐*N*’‐thiazol‐2‐yl‐hydrazines^196^ and 5‐Phenylamino‐2H‐[1,2,4] triazin‐3‐ones^197^ were successfully synthesized as promising inhibitors of *Mtb*LeuRS. However, further elucidation of the structure–activity relationships between these molecules and their targets is necessary to gain a deeper understanding of the inhibitory mechanisms and potential structural optimization of these molecules.

#### 3.3.3 Caseinolytic protease P

ClpP represents an ATP‐dependent, unfolding peptidase protein vital in preserving cellular homeostasis by degrading damaged and misfolded proteins. The joint expression of *Mtb*’s two *clpP* genes (ClpP1 and ClpP2) generates an active structure capable of hydrolyzing oligopeptides. However, aside from ClpP, ClpX, or ClpC1 is also necessary for the efficient hydrolysis of large, globular proteins.^198^ Candidate compounds can selectively bind to either the catalytic active center or the chaperone‐binding site of ClpP, while also partially influencing ClpC1 and ultimately affecting ClpP activity.^199^

β‐lactone derivatives, such as EZ120, represent promising inhibitors of ClpP, although further optimization is necessary to enhance their efficacy.^200^ The Bortezomib analog Pyr‐FL‐CMK displays *Mtb*ClpP selective inhibitory activity.^201^ Acyldepsipeptide antibiotics target the ATP‐binding site to curb the activity of ClpP,^202^ thereby stunting *Mtb* growth. In the same way, pyrrole derivatives are also targeted‐ClpP1P2 regulators.^203^ Lassomycin, a cyclic peptide synthesized by ribosomes, as well as ecumicin, a macrocyclic tridecapeptide, eradicate *Mtb* by targeting the ClpC1 ATPase complex.^204^, ^205^ Additionally, cyclomarin A serves to excessively activate ClpC1, thereby interfering with the usual function of ClpP,^206^ eventually resulting in the development of novel strategies for regulating ClpP activity.

#### 3.3.4 Proteasome

*Mtb* has a proteasomal degradation system that is responsible for the prompt degradation of the majority of damaged proteins. In this system, the prokaryotic ubiquitin‐like protein (Pup) plays the role of ubiquitin in the degradation process.^207^ *Mtb* proteasomes consists of 1 α and 1 β subunit, encoded by genes *prcA* and *prcB*, respectively.^208^ Peptidyl boronates, macrocyclic peptides, and phenylimidazole derivatives were reported as inhibitors of the proteasome.^208^, ^209^, ^210^ Previous studies have shown that deamidase of Pup (Dop) deamidates the C‐terminal glutamine of Pup to glutamate, preparing it for ligation to target proteins by proteasome accessory factor A (PafA).^211^ PafA can efficiently move Pup from one proteasome substrate, inositol 1‐phosphate synthetase, to two different proteins, malonyl‐CoA:ACP transacylase (FabD) and lonely guy (LOG).^212^ We recently developed a mutant of *Mtb*PafA, purified active PafA on a large scale, and conducted HTS to identify two promising PafA inhibitors, ST1926 and bithionol.^213^ In addition, the computational approach also yields potential inhibitors of the proteasome.^214^, ^215^

### 3.4 Amino acid synthesis and metabolism

The levels of various amino acids vary throughout distinct stages of infection and disease progression, reflecting the dynamic nature of infection.^216^ Amino acid synthesis and metabolic processes represent crucial determinants in *Mtb* survival and pathogenesis, underscoring their significance in combatting TB. This section describes the Ser/Thr protein kinases (STPKs) that regulate glutamate metabolism, the shikimate pathway involved in aromatic amino acid synthesis and the tryptophan synthase (TrpAB) involved in L‐tryptophan synthesis.

#### 3.4.1 Ser/Thr protein kinases

STPKs are phosphorylating enzymes that play essential roles in regulating various cellular functions. Within *Mtb*, 11 STPKs have been identified, of which PknA, B, and G (protein kinases A, B, and G) are indispensable for survival. Studies have highlighted the limited effectiveness of PknA and PknB inhibitors in targeting TB.^107^ Our recent work reports a series of antitubercular compounds based on ceritinib derivatives LPX‐16j,^217^ of which 5a has good efficacy and safety profile. The differential scanning fluorescence, isothermal titration calorimetry and molecular docking assays suggest that PknB may be one of the targets of 5a.^218^ Notably, PknG represents a vital kinase regulating glutamate metabolism.^219^ The inhibition of PknG kinase activity was first reported in vitro with the use of AX20017, a pioneering tetrahydrobenzothiophene compound that was specifically designed for this purpose.^220^ The inhibition of PknG activity has also been observed with nitro‐fatty acids,^221^ sclerotiorin,^222^ and steroidal lactones,^223^ which have all been reported as effective inhibitors of this kinase. NU‐6027, a dual inhibitor of PknG and PknD, has emerged as a potent inhibitor of *Mtb* growth in macrophages and mouse tissues.^224^ In recent studies, it has been suggested that PknG may additionally block autophagic flux by inhibiting phagosome maturation.^225^

#### 3.4.2 Shikimate pathway

The shikimate pathway comprises seven critical enzymatic steps, which are fundamental in *Mtb* and are connected with the synthesis of vital aromatic molecules^226^ (e.g., tryptophan^227^). Among these, inhibitors 3‐dehydroquinate synthase (DHQS), shikimate dehydrogenase, and shikimate kinase have been specifically targeted and have demonstrated noteworthy activity against *Mtb*. One of the important targets of multitarget compound IMB‐T130 is DHQS, which can effectively inhibit *Mtb*.^228^ IMB‐SD62, a lead triazolothiadiazole, and its derivatives were identified as inhibitors of shikimate dehydrogenase with antitubercular activity.^229^, ^230^, ^231^ Shikimate kinase inhibitor Compound 5631296, which was acquired through a comprehensive screening process, has demonstrated a remarkably low toxicity to HepG2 cells. Furthermore, it has exhibited synergistic activity when combined with RIF, resulting in the effective eradication of *Mtb*.^232^

#### 3.4.3 Tryptophan synthase

The indispensability of TrpAB for the sustenance of *Mtb* within macrophages and circumvention of host immune milieu renders it a highly auspicious therapeutic target.^227^, ^233^ In bacteria, fungi, and plants, the TrpAB bifunctional enzyme catalyzes the ultimate two steps of tryptophan biosynthesis and modulates pyridoxal 5′‐phosphate as an indispensable cofactor.^234^, ^235^, ^236^ TrpA converts indole‐3‐glycerol phosphate into glyceraldehyde‐3‐phosphate and indole. TrpB catalyzes PLP‐dependent β‐replacement reaction in which indole displaces the hydroxyl group of l‐Ser to produce l‐Trp.^237^, ^238^ An allosteric, mixed‐type inhibitor BRD4592 inhibits enzyme subunits and shows in vitro antitubercular efficacy.^233^ The same group also reported GSK1 and GSK2, which were found to target TrpAB in 2017 by Abrahams et al.,^239^ both bind to TrpAB very similarly to BRD4592.^238^

### 3.5 Nucleotide synthesis

*Mtb* must execute conserved DNA replication to transmit genetic information, a highly regulated process that represents a rich source of potential drug targets. This section describes the topoisomerase, the RNA polymerase (RNAP), and the transcriptional repressor of *ethA* (EthR).

#### 3.5.1 Topoisomerase

The genome of *Mtb* encodes a solitary type I topoisomerase (topoI) and a single type II topoisomerase (gyrase), comprising gyrA (*Rv0006*) and gyrB (*Rv0005*).^133^ Biochemical studies utilizing monoclonal antibodies and oligonucleotides have specifically demonstrated the site‐specificity of *Mtb*topoI.^240^ Various compounds have demonstrated inhibitory activity against *Mtb*topoI, including m‐AMSA,^241^ polyamines,^242^ imipramine, and norclomipramine,^243^ but appear limited in their cytostatic abilities. In comparison, DNA gyrase has emerged as a promising drug target for anti‐TB drug development. Several fluoroquinolone derivatives (Group A) have exhibited substantial inhibitory potential against TB, and are currently undergoing evaluation for the treatment of MDR‐TB and XDR‐TB.^1^, ^244^ X‐ray crystallography has proven to be an instrumental tool in the concerted effort to comprehend the precise mechanism by which fluoroquinolones affect DNA gyrase and to develop novel inhibitors for this crucial enzyme.^245^ In sharp contrast to fluoroquinolones—which chiefly target the N‐terminal domain of GyrA along with the C‐terminal domain of GyrB fused to GyrA—the newly developed phase II drug, SPR720,^246^ selectively targets GyrB. This aminobenzimidazole is both structurally and mechanistically dissimilar to fluoroquinolones, thus significantly reducing the risk of cross‐resistance. Thiazolopyridine ureas,^247^ thiazole‐aminopiperidine hybrid analogues,^248^ substituted benzofurans,^249^ and 4‐aminoquinolines^250^ have shown promising results as GyrB inhibitors with anti‐TB activity. Undoubtedly, the triumph of fluoroquinolones and the existence of other potential ligand‐binding sites^251^ in topoisomerase clearly suggest that the search for new topoisomerase inhibitors is a worthwhile scientific pursuit.

#### 3.5.2 RNA polymerase

RNAP is an evolutionarily conserved enzyme that plays a vital role in both transcription initiation and RNA elongation, and is subject to diverse regulatory mechanisms mediated by multiple transcription factors. In the case of *Mtb*, the RNAP is comprised of a central core that consists of five subunits (α2ββ’ω), with the β subunit being susceptible to inhibition by RIF.^252^ Several RIF analogues (e.g. rifamycin, rifalazil, and rifabutin) have been developed with the aim of enhancing the therapeutic efficacy of RIF through the same mechanistic pathway. Regrettably, a significant number of RIF‐resistant TB cases have emerged in clinical settings. Gene mutations that arise in *rpoB* are the primary culprits behind this phenomenon, rendering RNAP an unviable target for novel drug development.^253^, ^254^ Nevertheless, strategies aimed at targeting other critical transcriptional processes continue to be promising avenues for future investigations.^255^

#### 3.5.3 Transcriptional repressor of *ethA*

EthR is a repressor of *ethA*, a gene encoding a monooxygenase required for the activation of the prodrug ETH. Overexpression of *ethR*, which codes for the repressor EthR belonging to the TetR/CamR family of transcriptional regulators, has been found to induce potent inhibition of *ethA*.^256^ As previously mentioned, various thiocarbamide‐containing drugs, including ETH, rely on the activity of the monooxygenase EthA for activation. A breakthrough inhibitor of EthR, BVL‐GSK098, was developed via a combination of molecular design, screening, and optimization. This compound demonstrated impressive synergy with ETH combination therapy, as evidenced by a mouse model of TB.^257^, ^258^ Notably, the molecular targeting of EthR presents a groundbreaking approach that may help reverse ETH‐induced resistance. Spiroisoxazoline analogues,^258^ oxadiazole derivatives,^259^ and *N*‐phenylphenoxyacetamides^260^ have been discovered to possess EthR‐inhibitory and ETH‐enhancing properties. It is worth noting that first‐line drugs such as INH also require mycobacterial enzyme activation, making the development of transcriptional regulator‐targeting agents a pressing clinical need, as drug resistance often arises at this stage.

### 3.6 Energy metabolism

In recent years, significant attention has been devoted to *Mtb*’s energy metabolism—particularly, the oxidative phosphorylation pathway—with the aim of identifying novel strategies for pathogen control and drug discovery. Among these promising strategies are classes of antibacterial agents that target different elements of the oxidative phosphorylation pathway, which have shown significant efficacy in controlling dormant or latent mycobacterial infections. These novel therapeutic approaches hold tremendous potential for shortening the chemotherapy regimen for TB. In oxidative phosphorylation, the respiratory chain protein complexes facilitate the generation of a proton motive force (PMF) across a biomembrane, which is then harnessed by ATP synthase to produce ATP.^261^ This process involves several key steps, including (a) the transfer of electrons from NADH via the type II NADH dehydrogenase (NDH‐2) into the electron transport chain and (b) the acceptance of electrons by oxygen via a supercomplex comprising the cytochrome *bc*_1_ complex and the cytochrome *aa*_3_‐type terminal oxidase. Additionally, a cytochrome *bd*‐type terminal oxidase can directly accept electrons from the menaquinone pool.^262^

Clofazimine^263^ (Group B), a phase III drug currently used to target NDH‐2 in leprosy, is also undergoing repurposing as a treatment for TB. Additionally, clinical trials (phase I) have been initiated for TBI‐166,^264^ a riminophenazine analogue that may enhance the efficacy of clofazimine while reducing potential side effects. However, recent findings suggest that the activity of these drugs is not solely dependent on NDH‐2.^265^ Other agents that inhibit NDH‐2 and are commonly employed for the treatment of psychiatric disorders—such as thioridazine^266^ and other phenothiazines^267^—are currently being assessed as alternatives to conventional anti‐TB therapy. Telacebec (Q203), an imidazopyridine amide that targets the QcrB subunit of respiratory cytochrome *bc*_1_ complex, disrupts ATP synthesis. Encouraging results from phase I clinical trials regarding safety and tolerability have led to the initiation of phase II clinical trials examining the efficacy of Telacebec against MDR‐TB and XDR‐TB strains.^268^, ^269^ Another drug, PZA, affects PMF^270^ and is commonly used in combination with other respiratory chain inhibitors. Although quinazoline derivatives,^271^ morpholino thiophenes,^272^ arylvinylpiperazine amides,^273^ heterobiaryl side chain analogues,^274^ and imidazo[2,1‐b]thiazole derivatives^275^ have shown promise as inhibitors of QcrB, their metabolic stability requires further optimization.

#### 3.6.1 ATP synthase

ATP synthase is another crucial factor in *Mtb*’s energy metabolism and, as such, represents a critical target for drug development. Bedaquiline (BDQ)^276^, ^277^, ^278^ (Group A), a diarylquinoline compound, exerts potent antimycobacterial activity by binding to the c and Ɛ subunits of F‐ATP synthase, leading to the blockade of its proton pumping function. BDQ has been granted approval by the US FDA as a crucial component of short‐term XDR‐TB treatment regimens (BPaL regimen).^279^ This achievement confirms that ATP synthesis is a prime vulnerability in *Mtb* and that impairing the energy metabolism holds significant promise for shortening the duration of TB treatment. The successful initiation of phase I clinical trials for two dialkoxypyridine analogues^280^ (TBAJ‐876 and TBAJ‐587), which exhibit higher potency, significantly reduced lipophilicity, and pose a lower risk of cardiotoxicity, is a notable breakthrough following the clinical triumph of BDQ. Sudapyridine (WX‐081), a novel compound displaying similar efficacy to BDQ in the TB mouse model, boasts superior pharmacokinetic and toxicological profiles when compared with BDQ. WX‐081 is currently undergoing investigations in phase 2 clinical trials involving patients.^281^ In addition, squaramides^282^ and pyrazolopyrimidines^283^ are being investigated in preclinical studies.

### 3.7 Virulence

*Mtb* is an opportunistic slow‐growing intracellular organism whose multifaceted virulence mechanisms support the establishment of infection, persistence and reactivation.^284^ Consequently, efforts to develop *Mtb* virulence inhibitors are gaining increasing attention as a potential avenue for advancing TB control programs. This section describes the two‐component system (TCS) and the ESX‐1 secretion system of *Mtb*.

#### 3.7.1 Two‐component system

The TCS, a key pathogen–host signaling pathway constituted by two proteins responsible for transducing environmental cues into physiological responses, has emerged as a potent target for TB therapy. The canonical two‐component signaling pathway is comprised of a sensor kinase (SK) that detects specific environmental cues, and a cognate response regulator (RR) that mobilizes the necessary biological response in return.^285^ Several TCSs, including PhoPR,^286^ DosRST,^287^, ^288^ PdtaRS,^289^ and MtrAB,^290^ have demonstrated significant contributions to in vivo virulence and are, therefore, particularly attractive targets for future TB drug development.

##### PhoPR

PhoPR is recognized as a central regulator of pathogenic traits in MTBC strains, influencing the secretion of the virulence factor ESAT‐6, biosynthesis of acyltrehalose‐based lipids, and modulation of antigen export.^291^ Studies have revealed that low pH causes PhoPR phosphorylation, which, in turn, triggers the activation of the cytosolic redox sensor WhiB3.^292^ Given its role in *Mtb* virulence, PhoPR represents a compelling target candidate for TB therapy.^293^ Ethoxzolamide, a drug commonly used to manage duodenal ulcers, has been shown to inhibit PhoPR and significantly reduce *Mtb* load in both infected macrophages and mice.^294^

##### DosRST

DosRS was initially discovered to play a critical role in the survival and virulence of Mycobacterial spp. under hypoxic conditions.^295^, ^296^ Another SK, DosT, also contributes to sensing hypoxia and NO, alongside DosRS.^297^ *Mtb* exploits DosRST to establish and maintain nonreplicating persistence in response to hypoxia, NO, acid stress, or starvation.^298^ A recent whole‐cell phenotypic HTS campaign identified three inhibitors of DosRST, including artemisinin, HC102A, and HC103A. Artemisinin functions by disrupting heme‐based SKs DosS and DosT via the oxidation of ferrous heme, and subsequent heme‐artemisinin adduct formation. In contrast, HC102A and HC103A do not regulate DosS/T heme, but have been found to inhibit SK autophosphorylation.^299^, ^300^

#### 3.7.2 ESX‐1 secretion system

ESAT‐6 secretion system‐1 (ESX‐1) is a sophisticated type VII secretion system which is encoded by the RD1 locus.^301^ Its primary function is to facilitate the secretion of a variety of substrates such as ESAT‐6, EsxA, and EspB, among others, with the ultimate goal of inducing macrophage lysis.^302^, ^303^, ^304^ In addition, recent studies have proven that PhoPR inhibitors are highly effective in regulating ESX‐1 due to the fact that PhoPR serves as an essential mediator in activating ESX‐1 secretion.^305^ Leveraging the EsxA‐dependent cytolytic activity of *Mtb*, HTS has yielded two promising compounds. Notably, BBH7 and BTP15 not only significantly reduce intracellular bacterial load, but also promote phagolysosomal fusion in *Mtb*‐infected THP‐1 macrophages.^306^ More recently, HTS has produced new lead compound 3,5‐dinitrobenzamide.^307^

### 3.8 Others

#### 3.8.1 Folic acid and mycobactin biosynthesis

##### Dihydrofolate reductase

At the core of the folate pathway lies the pivotal role of dihydrofolate reductase (DHFR), responsible for catalyzing the transformation of dihydrofolate (DHF) into tetrahydrofolate (THF) using NADPH as an electron donor. Notably, existing DHFR inhibitors have shown limited efficacy against *Mtb*DHFR or are only weakly effective in inhibiting *Mtb*. However, *P*‐aminosalicylic acid (PAS), classified as a Group C drug, serves a dual role as both a substrate and prodrug within the folate pathway, with DHFR serving as one of its key targets.^308^, ^309^ Further analysis of the mechanism of PAS’ antifolate action has highlighted the potential benefits of utilizing compounds that can target multiple targets within the same pathway, thereby simplifying treatment regimens.^310^ Our team acquired the DHFR inhibitor AF‐353 through virtual screening and confirmed its selectivity between *Mtb*DHFR and hDHFR.^311^ In addition, we has predicted that ceritinib, a classical antilung cancer drug and its derivatives, may hold significant promise in combatting *Mtb* by serving as an effective DHFR inhibitor.^217^

#### 3.8.2 Metal uptake

##### Iron‐dependent regulator

In response to the stress imposed by the host, *Mtb* employs iron chelators known as siderophores, notably the mycobactin, to acquire iron. Meanwhile, *Mtb* has developed highly sophisticated intracellular iron sensing mechanisms, which are tightly regulated by the Fur (ferric uptake regulator) or DtxR (diphtheria toxin regulator) families.^312^ Among these, the mycobacterial iron‐dependent regulator (IdeR), a crucial metal binding transcriptional regulator of the DtxR family, plays a key role in maintaining mycobacterial iron homeostasis and facilitating virulence.^313^ Through virtual screening, numerous IdeR inhibitors, including acid alizarin violet N derivatives^314^ and short peptides,^315^ have been identified, though further analysis pertaining to structure–activity relationships is necessary to identify even more potent candidates.

#### 3.8.3 Cholesterol metabolism

##### Adenylyl cyclase

GSK2556286 (GSK‐286, phase I) is a pyrimidine‐2,4‐dione derivative that was uncovered from a HTS library of *Mtb*‐infected macrophages.^316^ Intriguingly, several in‐depth analyses of the *Mtb* survival cycle have revealed that cholesterol metabolism plays a pivotal role in facilitating *Mtb*’s survival within macrophages.^317^ In fact, in the absence of cholesterol utilization, *Mtb* is unable to establish an effective infection in macrophages and cannot effectively elicit pathogenesis.^318^ Notably, the *Mtb*AC possesses a specific ATP pocket that is distinct from its mammalian counterpart, thus playing a key role in converting NTPs into respective 3′,5′‐cyclic nucleoside monophosphates.^319^ Through direct adenylyl cyclase (AC) activation, GSK‐286 induces the generation of high levels of 3′,5′‐cyclic AMP (cAMP), thereby disrupting the *Mtb* cAMP signaling network.^320^ Furthermore, the agonist V‐58 is known to operate via a similar mechanism, thereby modulating cAMP signaling and inhibiting cholesterol metabolism by *Mtb*.^321^ It is worth noting that cAMP signaling is multifaceted in terms of its impact on *Mtb* pathogenesis, with studies indicating that it can regulate TNF‐α by macrophages.^322^

**FIGURE 4:**
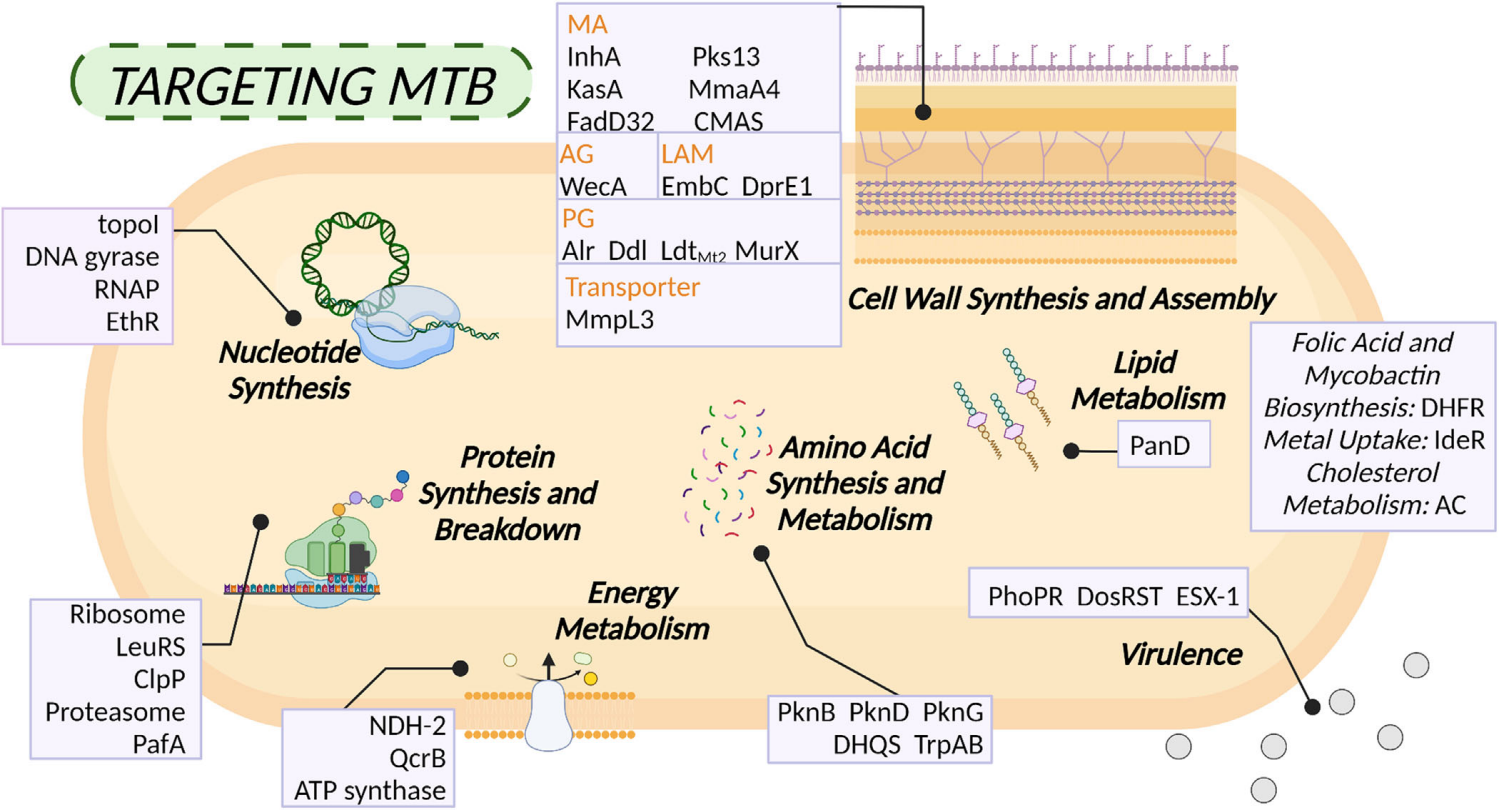
Overview of antituberculosis targets aimed at *Mtb*. Disruption of crucial pathways in *Mtb*, such as cell wall synthesis and assembly, protein synthesis and breakdown, and energy metabolism, has been regarded as a potent strategy for combating tuberculosis. Recently, there has been a growing interest in interventions focused on lipid metabolism, amino acid synthesis and metabolism, nucleotide synthesis and virulence. The elements in the figure were drawn using BioRender online tool (https://biorender.com).

Figure 4 provides a comprehensive summary of the main targets of anti‐TB compounds targeting pathogens. Indirect effects of GSK‐286 and V‐58 on host macrophages get us thinking. Notably, the interaction between pathogens and their host is a crucial factor in determining bacterial pathogenicity and virulence. It is true that all drugs in the current clinical pipeline target the pathogen directly. In fact, by targeting cell wall synthesis and assembly, protein synthesis, nucleotide synthesis, energy metabolism, folic acid, and cholesterol metabolic pathways, existing drugs have already shown great promise in achieving high clinical outcomes.^323^ Current trends in drug development involve bi‐directional screening for both cellular and target‐based activity, with a focus on multitarget candidates. Finding new targets and their inhibitors in *Mtb* remains a promising strategy for combating drug resistance and developing potent lead molecules, though it is important to acknowledge the potential drawbacks such as the lengthy investment required for development and their limitations in treating both active and latent TB. Due to these challenges, an increasing number of studies have recently devoted their efforts towards host‐directed therapies for TB treatment.

## 4 HOST‐DIRECTED THERAPY

The infection process of *Mtb* is highly dependent on host cells and requires the utilization of multiple strategies to persist within infected cells. Although host‐directed therapy (HDT) is often viewed as an adjunctive regimen, recent clinical studies have shown that it can lead to rapid anti‐TB effects and improved prognosis.^324^ The complex immune events that occur during *Mtb* infection and pathogenesis offer numerous opportunities for HDT, and ongoing discoveries pertaining to the involved pathways and molecular participants continue to expand the list of potential molecules that can serve as anti‐TB treatments. Currently, TB HDT strategies are focused on three main objectives: (a) enhancing host immune defense, (b) interfering with the use of host mechanism, and (c) limiting immunopathology.

### 4.1 Enhancing host immune defense

Innate immune responses include cells and mechanisms that are either constantly present or are activated within minutes to hours following an infection to suppress the replication and spread of the invading *Mtb*. Multiple immune cells are endowed with a repertoire of pattern recognition receptors, including TLRs, NLRs, and CLRs, each of which has been implicated in the recognition and internalization of *Mtb*.^325^, ^326^ These innate mechanisms act as a first line of defense against pathogenic microorganisms and are essential in shaping the subsequent adaptive immune response.^327^ Phagocytic DCs present *Mtb* antigen to T lymphocytes and promote activation and differentiation of naïve CD4 T cells and naïve CD8 T cells.^328^, ^329^ This process necessitates the presentation of antigen in the context of major histocompatibility complex (MHC), costimulatory molecules, and the necessary cytokines.^11^

The immunity‐centered HDT approach emphasizes the development of IFN‐α and IFN‐γ supplementation regimens, which, when utilized in conjunction with antimycobacterial therapy, may potentially influence the progression of pulmonary TB.^330^, ^331^, ^332^ Nonetheless, we are predominantly concerned with regulating the abundance of them to influence the immune process. Several methods have been devised to enhance the endogenous IFN response, depending mainly on the activation of TLRs, but their use in the treatment of TB has not been reported.^333^, ^334^ Metformin (MET), the most widely administered diabetes drug, has been proposed as a candidate adjunctive HDT for TB.^335^ In humans, MET exhibits a multitude of effects, such as the production of TNF‐α, IFN‐γ, and interleukin 1β (IL‐1β), augmented phagocytosis activity, and increased production of ROS.^336^ Myeloid‐derived suppressor cells (MDSCs) are increasingly recognized as a critical driver of TB pathogenesis and represent an immunosuppressive cell population.^337^ In fact, elevated levels of MDSCs have been observed in both blood and sputum of patients with active TB in studies,^338^ and have also been induced in healthy individuals after exposure to *Mtb*.^339^ These cells play a detrimental role in diminishing protective T‐cell responses and may contribute to the inability of hosts to eradicate the infection, which subsequently leads to the development of TB disease.^340^ Tasquinimod,^341^ an experimental quinoline‐3‐carboxamide, has demonstrated tremendous promise in inhibiting tumor growth in murine cancer models and has recently been shown to deplete MDSCs and reduce the relative bacterial burden in the lung and spleen of murine models of TB^342^ (Table 2).

**TABLE 2:** Targets, drug candidates, and actions of host‐directed therapeutics for tuberculosis.

Effect of HDT		Target	Typical compound	Mechanism of action	Drug stage for TB
Enhancing host immune defense		Myeloid‐derived suppressor cells (MDSCs)	Tasquinimod^341^	Antagonizing MDSCs to activate T cells	Biological test
		Tyrosine kinase (TK)	Imatinib^348^	Inhibition of TK to activate phagolysosomal acidification	Phase II
		Ca2+‐adenosine monophosphate‐activated protein kinase (AMPK)	GABA^349^	Inhibition of Ca2+‐AMPK to enhance phagosomal maturation	Biological test
		Indoleamine 2,3‐dioxygenase (IDO)	D‐1MT^355^	Inhibition of IDO to increase CD4 T cells	Biological test
		Heme oxygenase‐1 (HO‐1)	SnPPIX^356^	Inhibition of HO‐1 to increase iNOS and NO production	Biological test
		Histone deacetylases (HDACs)	Trichostatin A^362^	Inhibition of HDAC6 to modify epigenetics	Biological test
Interfering with the use of host mechanism	Regulation of autophagy	Mammalian target of rapamycin (mTOR)/AMPK	Rapamycin^375^	Regulation of mTOR/AMPK to enhance autophagy	Biological test
		Extracellular regulated protein kinase ERK1/2	Pasakbumin A^393^	Activating ERK1/2 to enhance autophagy	Biological test
		Epidermal growth factor receptor (EGFR)	Gefitinib^399^	Inhibition of STAT3 to enhance autophagy	Biological test
		Tyrosine kinase (TK)	Ibrutinib^401^	Inhibition of BTK/Akt/mTOR to enhance autophagy	Biological test
		Transcription factor EB (TFEB)	Statins^403^, ^404^, ^405^	Activating AMPK/mTORC1/TFEB to enhance autophagy	Biological test
		Sirtuin 1/3 (SIRT1/3)	Resveratrol^406^	Activation of SIRT1/3 to enhance autophagy	Biological test
		Toll‐like receptor 7 (TLR7)	Imiquimod^409^	Agonism of TLR7 to enhance autophagy	Biological test
	Intervention of granuloma formation	Vascular endothelial growth factor (VEGF)	Pazopanib^415^	Inhibition of VEGF to impede granuloma angiogenesis	Biological test
		Angiopoietin‐2 (ANG‐2)	AKB‐9778^418^	Inhibition of ANG‐2/TIE2/VE‐PTP to reduce infection‐induced vascular permeability	Biological test
	Regulation of cell death	Myeloid cell leukemia sequence‐1 (Mcl‐1)	Sabutoclax^443^	Inhibition of Mcl‐1 to activate apoptosis	Biological test
		Complex I	Metformin^396^	Inhibition of complex I to reduce necrosis	Phase II
		Ferroptosis	Ferrostatin‐1^446^	Inhibition of ferroptosis to reduce necrosis	Biological test
		Mitogen‐activated protein kinase (MAPK)	Corticosteroids^447^	Inhibition of p38 MAPK to reduce necrosis	Phase II
		Domain‐like receptor protein 3 (NLRP3)	Baicalin^453^	Inhibition of PERK/TXNIP/NLRP3 to reduce pyroptosis	Biological test
Limiting immunopathology		Matrix metalloproteinase (MMP)	Doxycycline^466^	Inhibition of MMP to reduce cavitary pathology	Phase III
		5‐Lipoxygenase (5‐LOX)	Zileuton^477^	Inhibition of 5‐LOX to regulate lipid metabolism	Biological test
		Cyclooxygenase‐2 (COX‐2)	NSAIDs^481^	Inhibition of COX‐2 to reduce inflammatory response	Phase II
		Glucocorticoid receptor (GR)	Corticosteroids^484^	Binding to GR to reduce inflammatory response	Phase II
		Poly(ADP‐ribose) polymerase 1 (PARP1),	PJ‐34^487^	Inhibition of PARP1 to reduce inflammatory response	Biological test
		Phosphodiesterase‐4 (PDE‐4)	Dovramilast^488^	Inhibition of PDE‐4 to reduce inflammatory response	Phase II

Notably, studies have shown that enhancing phagocytosis can effectively limit the intracellular growth of *Mtb*.^343^, ^344^ Phagocytosis serves as the fundamental link between the innate and adaptive branches of the immune system.^345^ In addition to effectively isolating and eliminating pathogens, the phagocytic process also plays a critical role in triggering the activation of adaptive immune responses. Following the ingestion of pathogens, phagosomes must undergo a sequence of dynamic transformations involving both the membrane and internal components, ultimately allowing for their maturation and subsequent fusion with lysosomes.^346^ Pathogens frequently resort to various cunning strategies to evade capture, including evading detection, disrupting signaling pathways, or disabling the machinery that drives the phagocytic process.^347^ Imatinib, a chemical inhibitor of tyrosine kinase (TK), has been found to promote *Mtb* killing through the activation of cathepsin D and phagolysosomal acidification.^348^ Gamma‐aminobutyric acid, an inhibitory neurotransmitter, is known to regulate the Ca2+‐AMPK (adenosine monophosphate‐activated protein kinase) pathway, thereby enhancing phagosomal maturation.^349^

In the context of adaptive immune responses, antigen‐presenting cells (APCs) play a pivotal role in phagocytosing antigens and attaching them to MHC class I or II molecules, thereby presenting the antigens to T cells to initiate adaptive T cell responses. Facilitating the proper activation of APCs is an effective strategy to help hosts recognize *Mtb*. G1‐4A, a polysaccharide derived from *Tinospora cordifolia* and a reported TLR inhibitor, has shown promising results in improving host defense against *Mtb*. In fact, G1‐4A has been found to increase NO and proinflammatory cytokine secretion (such as TNF‐α, IL‐β, IL‐6, IL‐12, IFN‐γ) through upregulating MHC‐II, thus leading to reduced intracellular survival of *Mtb*.^350^ Additionally, vitamin D, which is required for TLR production, has been recognized as a key molecule in host defense against TB.^351^ However, more research is necessary to fully elucidate the role of vitamin D in the prevention and treatment of TB.^352^ In fact, *Mtb* devotes considerable energy to directing the induction of the cellular response to infection.^353^ *Mtb* induces the expression of indoleamine 2,3‐dioxygenase (IDO), which degrades tryptophan and attenuates T cell and NK cell proliferation to suppress immunity.^354^ The specific inhibitor of IDO activity, D‐1MT, has been shown to improve clinical outcomes by increasing the entry of CD4 T cells into granulomas.^355^ Heme oxygenase‐1 (HO‐1), an antioxidant enzyme, is induced by *Mtb* to be expressed in the lung. The inhibition of HO‐1 activity with tin protoporphyrin XI (SnPPIX) was found to enhance iNOS expression and NO production by *Mtb*‐infected macrophages following activation by IFN‐γ produced by T lymphocytes, consequently allowing for a more efficient control of bacterial replication by host cells.^356^

The epigenetic changes elicited by *Mtb* infection play a pivotal role in circumventing the immune response of the host and thereby inducing bacterial persistence and dissemination.^357^ Epigenetic modifications used by *Mtb* to evade host immune responses include histone acetylation,^358^ noncoding RNA expression^359^, ^360^ and DNA methylation.^361^ Targeting epigenetics works have been undertaken for the management of TB. Studies have shown that Trichostatin A impedes host histone deacetylases (HDACs) and, as a result, augments both in vitro and in vivo antimycobacterial efficacy in human macrophages.^362^ Tubastatin A, an inhibitor of HDAC6, fortifies the immune response and curbs the growth of mycobacteria in an *Mtb*‐infected mouse model.^363^ Valproic acid and suberoylanilide hydroxamic acid (vorinostat), both inhibitors of HDACs, have supplementary potential to INH and RIF regimens.^364^ Also as an adjunct to standard TB treatment, the combination of 4‐phenyl butyrate (a nonspecific HDACs inhibitor) with vitamin D3 administered orally has shown beneficial effects on clinical recovery.^365^, ^366^ DNA methylation inhibitors, such as 5‐azacytidine, belong to another class of compounds that target host epigenetics.^367^ Bristol‐Myers Squibb has previously submitted a phase Ib/IIa open label, nonrandomized clinical trial to investigate whether the use of injectable azacitidine affects DNA methylation levels and immune signaling pathways during the treatment of pulmonary TB. Unfortunately this study has now been withdrawn.^368^ Developing methods to utilize small‐molecule drugs to influence the course of immune events is a promising avenue of research, especially given the fact that most of events are still being explored.

### 4.2 Interfering with the use of host mechanism

*Mtb* is an intracellular parasitic bacterium that relies on host cell mechanisms in order to proliferate and persist. Its replication and persistence is determined by a multitude of cellular processes, including autophagy, granuloma formation, and the specific type of cell death that occurs within infected cells (such as apoptosis, necrosis, and pyroptosis).^369^ The majority of novel HDT strategies operate by disrupting these processes, thereby inhibiting the survival potential of *Mtb*.

#### 4.2.1 Regulation of autophagy

Autophagy is a key mediator responsible for the degradation of damaged macromolecules and organelles. Autophagy is regulated primarily by the mammalian target of rapamycin (mTOR) complex 1 and the AMPK.^370^, ^371^

##### mTOR/AMPK

The 5′‐adenosine AMPK plays a crucial role in maintaining cellular material and energy homeostasis via phosphorylation.^372^ The mammalian target of mTOR is a serine/threonine kinase that operates through two distinct complexes (mTORC1 and mTORC2) and regulates cellular metabolism in response to environmental cues.^373^ Autophagic signaling is typically divided into mTOR‐independent and mTOR‐dependent pathways, with the latter serving as a negative regulatory pathway through which *Mtb* inhibits host autophagy.^374^ Inhibition of mTOR has therefore emerged as a viable strategy for counteracting the low autophagic state observed in *Mtb* infections. One classical example of an mTOR inhibitor is rapamycin, which is capable of inducing in situ autophagy in lung macrophages and has been found to effectively alleviate *Mtb* burden when utilized in conjunction with INH or RIF via inhalation. This effect is mediated by the phosphorylation of S6 kinase.^375^It is worth noting that the use of rapamycin is currently limited by its potential for immunosuppression.^376^ In contrast, the antiprotozoal drug nitazoxanide has been found to strongly stimulate autophagy while inhibiting mTOR signaling.^377^ Additionally, the antidepressant amoxapine has demonstrated the ability to induce autophagy and protect macrophages during infection.^378^ The anticonvulsant drugs carbamazepine and valproic acid, meanwhile, can induce mTOR‐independent autophagy through AMPK activation, a unique characteristic that sets them apart from other drugs.^379^ MET, the antidiabetic drug, has been reported to significantly reduce intracellular *Mtb* growth in an AMPK‐dependent manner.^380^

##### TNF‐α

The TNF‐α signaling pathway has proved amenable for therapy of autoimmune and other chronic inflammatory noninfectious diseases.^381^, ^382^ Multiple cells synthesize this cytokine in response to mycobacterial infection to induce a phagocytosis program,^383^, ^384^, ^385^, ^386^, ^387^ and the regulatory process involves IFN‐γ,^384^ enzymes,^388^ and lipid mediators.^389^ TNF‐deficient mice infected with *Mtb* exhibit delayed chemokine induction and immune cell recruitment.^390^ Maintaining its normal level is essential to activate autophagy.^391^ TNF‐α is also associated with granuloma biogenesis and integrity, driving the formation of durable solid granulomas.^392^ Since none of the approved TNF modulators are small‐molecule drugs, they are not discussed in this review. Pasakbumin A, which is extracted from *Eurycoma longifolia Jack*, effectively inhibits intracellular *Mtb* killing by inducing both autophagy and TNF‐α production through the extracellular regulated protein kinase ERK1/2‐ and nuclear factor NF‐κB‐mediated signaling pathways in *Mtb*‐infected cells.^393^ It is noteworthy to mention that the studies have reported a higher incidence of active TB among patients receiving TNF‐neutralizing therapy.^394^, ^395^ Notwithstanding its beneficial effects on granuloma formation, overexpression of TNF‐α can promote inflammation while contributing to the hyperactivation of infected macrophages in the granuloma and provoke programmed necrosis^396^, ^397^ (as detailed below).

##### Protein kinase

Kinases are central to mammalian signaling pathways. The screening process for identifying compounds that suppress the proliferation of *Mtb* in macrophages yielded two promising candidates, namely imatinib, which was previously mentioned, and gefitinib, an inhibitor of the epidermal growth factor receptor (EGFR).^398^ The therapeutic application of gefitinib in *Mtb*‐infected macrophages has been demonstrated to effectively restrain the STAT3 signaling pathway, a transcription factor that has been found to impede effective immune responses in vivo.^399^ Additionally, gefitinib treatment was observed to stimulate the expression of genes associated with lysosomal biogenesis and function, resulting in an increased production of functional lysosomes with enhanced autophagy.^400^ According to recent research, the TK inhibitor, ibrutinib, which is commonly used in the treatment of chronic lymphocytic leukemia, has been identified to have efficacy against *Mtb* infection by inhibiting the BTK/Akt/mTOR signaling pathway and inducing autophagy. This treatment has demonstrated a significant reduction in bacterial load in *Mtb*‐infected mice models.^401^

In addition to the aforementioned drugs, there is growing interest in the development of modulators that regulate autophagy through alternative pathways. Autophagy‐related proteins are regulated by the transcription factor EB (TFEB).^402^ Recent studies have demonstrated that statins can induce autophagy by altering cellular AMP:ATP ratios in *Mtb*‐infected macrophages and activating the AMPK/mTORC1/TFEB axis.^403^, ^404^, ^405^ Meanwhile, the activation of host sirtuin 1 (SIRT1) has also been shown to reduce intracellular growth of drug‐susceptible and drug‐resistant strains of *Mtb*, inducing phagosome‐lysosome fusion and autophagy through a SIRT1‐dependent mechanism.^406^ In recent studies, it has been demonstrated that the sirtuin 3 (SIRT3), coordinates mitochondrial function and autophagy activation to facilitate anti‐*Mtb* responses via peroxisome proliferator activated receptor alpha (PPARA).^407^ Natural activators such as resveratrol^406^ and honokiol^407^ activate SIRT1 and SIRT3, respectively, exerting their inhibitory effects on intracellular growth of *Mtb*. Additionally, the use of 4‐phenyl butyrate in combination with vitamin D shows potential in counteracting the inhibition of human antimicrobial peptide LL‐37 expression by bacilli, thereby promoting autophagy.^408^ This finding suggests that regulation of antimicrobial peptides could serve as an approach to promote autophagy.^408^ Imiquimod (IMQ), a TLR7 agonist, has been shown to act as a radiosensitizer for melanoma by inducing autophagy.^409^ Recent studies indicate that IMQ can stimulate TLR7 and activate autophagy by increasing the production of ROS through the p38‐ or MEK/ERK1/2‐mediated signaling pathways during the early phase.^410^ Further research has outlined the two E3‐Ubiquitin (E3‐Ub) ligases, PRKN and SMURF1, that specifically target phagophores by tagging poly‐Ub,^411^ highlighting the efficacy of targeting ubiquitin machinery in regulating autophagy.

#### 4.2.2 Intervention of granuloma formation

Pathogenic mycobacteria have been shown to induce the formation of complex cellular aggregates known as granulomas, which are a hallmark of TB. These granulomas are composed of various immune cells, including macrophages, DCs, and T cells, which surround and attempt to contain the mycobacteria through the formation of a barrier. However, the bacteria can persist within the granuloma, which can lead to recurrent or chronic infections. If uncontrolled, the formation of granulomas can lead to the development of active TB in human patients.^412^

##### Angiogenesis obstruction

In tumors, the hypoxic environment induces VEGF expression and stimulates angiogenesis.^413^ Significantly high levels of serum VEGF were also found in patients with active TB.^414^ In 2015, Oehlers et al. reported that intercepting VEGF pathway signaling by pazopanib (a VEGF receptor inhibitor^415^) or SU5416 (a prototypical TK receptor^416^) inhibits granuloma‐associated angiogenesis, reduces *Mtb* burden and limits the spread.^417^ Subsequent studies have demonstrated that angiopoietin‐2 (ANG‐2) expression is prominently upregulated within granulomas. Specifically, pharmacological inhibition of vascular endothelial‐protein tyrosine phosphatase^418^ (AKB‐9785, a structurally similar and equivalent inhibitor to AKB‐9778, patented from Aerpio Therapeutics^419^, ^420^) interrupts the ANG‐2/TIE2/VE‐PTP axis and severs the stabilizing effects of this pathway, culminating in vascular permeability alterations that effectively limit the growth of *Mtb*.^421^

##### Metabolism blockage

Previous studies have reviewed the strategies by which *Mtb* influences host‐pathogen interactions by regulating metabolism.^422^ The metabolic alterations culminating due to stressors present in the microenvironment of granuloma—namely a paucity of nutrients, hypoxia, and acidic pH—hold great significance. The metabolic transition from oxidative phosphorylation to aerobic glycolysis of cells in the presence of sufficient oxygen,^423^ a process known as the Warburg effect,^424^ is present in tumors^425^ and has parallels within TB granulomas.^426^ As a consequence, regulation of glycolysis, glucose transport and glucose homeostasis are important strategies to limit granuloma metabolism. Treatment to limit aerobic glycolysis can limit granuloma as well as *Mtb* replication. *Mtb*‐infected macrophages in granuloma undergo metabolic changes, including upregulation of hypoxia‐inducible factor‐1α (HIF‐1α) and using glutamine as an important carbon and nitrogen source.^427^, ^428^ Insufficient cytoplasmic aspartate delivery leads to cell death when TCA circulating carbon is reduced after glutamine inhibition.^429^ Foamy macrophages, a granuloma‐specific cell population characterized by its high lipid content, constitute a long‐term reservoir for *Mtb* in the human host.^430^ Lipid bodies serve as both a source of nutrients and a secure niche for the bacterium.^431^ Formation of *Mtb*‐dependent lipid bodies is mediated through the G protein‐coupled receptor GPR109A. Inhibition of the GPR109A leads to a reduced bacterial load and the reduction in alveolar macrophage lipid bodies in vivo, which are associated with granuloma caseation.^431^ The significance of glycolysis and lipid metabolism in the advancement of *Mtb* infection highlights the potential for HDT. In addition, metabolic alterations within granulomas can lead to the accumulation of advanced glycation end products (AGEs). These AGEs are known to affect macrophage death and activation, potentially playing a role in the progression of TB.^432^

#### 4.2.3 Regulation of cell death

Cell death can be classified into two main categories based on molecular mechanisms, morphological characteristics, and signal dependency: programmed cell death (PCD) and nonprogrammed cell death (n‐PCD).^433^ The interaction between *Mtb* and the host in relation to cell death is complex, with *Mtb* initially inhibiting host cell apoptosis to ensure replication niches, and subsequently inducing necroptosis and pyroptosis after successful replication to release and infect new cells.^434^

##### Apoptosis activation

Numerous studies^435^, ^436^ have demonstrated that *Mtb* has the ability to regulate proapoptotic proteins to inhibit the intrinsic pathway of host cell apoptosis, and this escape ability is thought to be a critical virulence factor.^437^ Currently, much work is being done to regulate antiapoptotic genes and controlling apoptotic factors with the aim of enhancing host cell apoptosis.^438^ Peroxisome proliferator‐activated receptor gamma (PPARγ), a member of the nuclear receptor superfamily, is a transcriptional factor that governs inflammation.^439^ This protein exhibits elevated expression within activated alveolar macrophages and macrophage‐derived foam cells, each of which plays a significant role in the pathogenesis of TB.^440^, ^441^ *Mtb* and its cell wall mannose‐capped lipoprotein mannan activate PPARγ and stimulate MRs to enhance their intracellular survival.^442^ To prevent apoptosis, *Mtb* differentially regulates the Bcl‐2 (B‐cell lymphoma protein 2) family members Bax (proapoptotic) and Mcl‐1 (prosurvival) expression through PPARγ.^443^ Therapeutics aimed at Mcl‐1, alongside other Bcl‐2 prosurvival proteins, is currently under development as potential cancer treatments.^444^ The Mcl‐1 inhibitors sabutoclax, TW‐37, A‐1210477, and MIM‐1 have been shown to significantly decrease *Mtb* survival within human macrophages via the activation of apoptosis.^443^ These promising results indicate the feasibility of Mcl‐1 and other antiapoptotic proteins as viable targets for HDT.

##### Necrosis suppression

Conversely, another idea that is being explored is the reversing of cell necrosis. The necrosis of infected macrophages represents a crucial event in the pathogenesis of TB as it results in the release of mycobacteria into the extracellular environment, which is permissive for the growth and spread of the pathogen. Excess TNF has been shown to trigger programmed necrosis.^397^ Recent research has elucidated the mechanism by which excess TNF induces mitochondrial ROS (mROS) production in TB. It has been found that excess TNF in macrophages infected with mycobacterium triggers an increase in mROS production through the reverse electron transport process, which occurs through complex I. MET may inhibit complex I and thus prevent TNF‐induced mROS and macrophage necrosis.^396^ Moreover, excess TNF‐α following infection has been found to upregulate mROS and induce the formation of the mitochondrial permeability transition pore complex, subsequently triggering necrosis via cyclophilin D (CypD). The inhibitory peptide alisporivir of CypD has been found to synergistically inhibit Mycobacterium zebrafish infection when used in combination with the ceramide inhibitor desipramine.^445^ Interestingly, it has been found that ceramide is induced to be produced by TNF‐α.^445^ Studies have demonstrated a significant correlation between *Mtb*‐induced host cell necrosis and ferroptosis, with decreased lung bacterial load observed in mice with acute TB infection following ferroptosis inhibitor, Ferrostatin‐1 treatment.^446^ Furthermore, corticosteroids have been found to inhibit necrosis by targeting mitochondrial membrane stability through the inhibition of p38 mitogen‐activated protein kinase (MAPK) in addition to their known actions through glucocorticoid receptors (GRs).^447^

##### Pyroptosis suppression

Pyroptosis is a proinflammatory PCD pathway distinct from other forms of cell death, mediated by pyroptosomes, dependent on N‐terminal fragment of the caspase‐1‐cleaved gasdermin D (GSDMD) and causes plasma membrane rupture.^448^, ^449^ Two known mechanisms for initiating pyroptosis are through (a) damage to the plasma membrane of host cell by the ESX‐1 system, which activates the NLRP3 (domain‐like receptor protein 3) inflammasome,^450^ and (b) interaction between EST12, a protein that induces cell pyroptosis, and active C kinase 1, leading to activation of the macrophage NLRP3.^451^ Ultimately, both mechanisms result in the activation of the inflammasome‐caspase‐1‐GSDMD pyroptosis‐IL‐1β immune pathway. Also, upon internalization in the phagosome, *Mtb* create phagosomal pores that allow for the passage of bacterial factors, ultimately activating the cGAS/STING pathway and resulting in an IFN‐γ response.^452^ Research shows that baicalin reduced pyroptosis by inhibiting the PERK/TXNIP/NLRP3 axis.^453^ Additionally, Tanshinone IIA inhibited upstream signals of NLRP3 inflammasome activation in *Mtb*‐infected macrophages.^454^ Despite this understanding of the mechanisms behind pyroptosis induction in *Mtb*, there are currently no known modulators for the pyroptosis suppression for the clearance of *Mtb*.

### 4.3 Limiting immunopathology

The immunopathological response seen in TB, characterized by excessive inflammation, can present a significant challenge in managing the disease.^455^ Research has confirmed that immune cells utilize distinct strategies to control *Mtb* invasion, with proinflammatory mechanisms playing a crucial role in slowing, sequestering, and ultimately eliminating the pathogen. The successful host response, however, relies on balancing the timing and expression levels of pro‐ and anti‐inflammatory responses.^456^ Consequently, regulation of inflammation to balance protective and pathological elements of the immune response is an integral component of HDT.

Cavitary pathology is associated with host‐protease imbalance driven by *Mtb*.^457^ Unbiased investigations conducted by various research groups, using a diversity of methodological approaches, have consistently identified matrix metalloproteinases (MMPs) as one of the most highly upregulated proteins in TB.^458^, ^459^, ^460^, ^461^, ^462^, ^463^, ^464^ MMPs depend on zinc ions to carry out the hydrolysis of protein substrates, and are highly associated with TB cavity in patients.^465^ The original ribosome inhibitor, doxycycline, has been found to decrease MMP activity in cellular models and suppress mycobacterial growth in vitro and in guinea pigs, as evidenced by clinical trials.^466^ Marimastat, an antitumor drug specifically designed to inhibit MMP, was not ultimately approved for use due to musculoskeletal toxicity, recent studies have demonstrated its efficacy in reducing granuloma formation and *Mtb* bacterial load when combined with RIF or INH.^467^ Other MMP inhibitors that have been reported to improve granuloma production are batimastat, prinomastat, Sb‐3ct,^467^ and some specific antibodies.^468^ However, the MMP‐7 inhibitor cipemastat increased the frequency of lung cavitation in mice with TB.^469^ Additional research is necessary to comprehend the potential of MMP inhibitors as supplementary therapeutic interventions for pulmonary TB.

Host lipid metabolism plays an essential role in nodule–host interactions.^470^ Lipid peroxidation, a process that causes oxidative damage to lipids in host cell membranes, has been found to induce tissue necrosis and facilitate the transmission of *Mtb*.^471^ In vivo experiments have shown that lung necrosis in acutely *Mtb*‐infected mice is associated with reduced glutathione peroxidase‐4 (Gpx4) expression as well as increased lipid peroxidation.^446^ *N*‐acetylcysteine restores the reduced form of glutathione and counteracts TB‐induced oxidative stress.^472^ The addition of *N*‐acetylcysteine to the standard drug regimens for TB has been demonstrated to increase the levels of glutathione peroxidase in TB patients.^473^ As a result of this finding, clinical trials assessing the efficacy of *N*‐acetylcysteine‐assisted treatment for TB patients are currently underway.^474^ Host eicosanoids, lipid mediators of inflammation, have been shown to respond to the severity of TB and TB‐diabetes comorbidity,^475^, ^476^ and have a significant impact on the outcome of *Mtb* infection through their correlation with two major counter‐regulatory classes of inflammatory cytokines: IL‐1 and type I IFNs.^477^ Currently, modulation of lipid metabolic pathways (5‐lipoxygenase, 5‐LOX modulators) has been reported for the treatment of TB infection. Zileuton, a 5‐LOX inhibitor originally developed for asthma, has been found to improve survival, reduce bacterial burden, and alleviate lung injury in *Mtb*‐infected mice.^477^ In oncology studies, cyclooxygenase‐2 (COX) has been shown to be associated with MDSC activation, and COX‐2 inhibitors such as aspirin and ibuprofen have been utilized to test their efficacy in treating mouse models of infection, where they demonstrated an increase in bacterial clearance when combined with PZA.^478^, ^479^ However, recent studies have reported that COX‐2 inhibitor treatment significantly reduces Type‐1 helper (Th1) differentiation and downregulates IFN‐γ expression, which exacerbates *Mtb* aerosol infection.^480^ As such, more experimental evidence is required to determine the appropriate role of nonsteroidal anti‐inflammatory drugs (NSAIDs) in adjuvant anti‐TB therapy.^481^

Corticosteroid therapy probably improves neurological outcomes of, and decreases mortality due to, tuberculous meningitis of moderate severity.^482^ Due to their potent anti‐inflammatory properties, these drugs are capable of suppressing the host's immune response, thereby reducing the potential for immunopathology. A recent study has demonstrated a differential expression of GR pathway‐regulated genes in patients with TB.^483^ Promisingly, the corticosteroids dexamethasone and prednisolone have shown efficacy in reducing mortality from all forms of TB, including pulmonary TB.^484^ A randomized, double‐blind, placebo‐controlled trial has demonstrated that the administration of prednisone is effective in reducing the need for hospitalization and therapeutic procedures, while also accelerating improvements in symptoms, performance, and quality of life.^485^

Poly(ADP‐ribose) Polymerase 1 (PARP1), a member of the PARP family and a regulatory factor, is known to induce further activation of itself through the actions of inflammatory mediators.^486^ In light of this, PARP1 inhibitors PJ‐34 have recently been proposed as an HDT approach to TB, due to their ability to reduce inflammation and alleviate lung disease.^456^, ^487^ The phosphodiesterase‐4 (PDE‐4) inhibitor Dovramilast (CC‐11050) has been found to effectively downregulate TNF‐α production in macrophages through the increase of intracellular cAMP, thereby reducing inflammation in rabbits with pulmonary TB during INH treatment.^488^ When administered in combination with INH, roflumilast, another PDE‐4 inhibitor, was shown to effectively decrease *Mtb* bacterial load in mice.^489^ Notably, the role of the Janus kinases (JAKs)/signal transducers and activators of transcription (STATs) pathway in the development of TB has received interest. The JAK/STAT pathway regulates multiple TB‐related cytokines, including IL‐27^490^ and IFN‐γ,^491^ affecting T cell subset differentiation, T cell activation and generation of memory.^492^ LINC00870, an upregulated long, noncoding RNAs in *Mtb*‐infected peripheral blood mononuclear cells (PBMCs), promoted p‐STAT5 and p‐JAK2 protein expression, thus activating JAK/STAT signaling in PBMCs.^493^ Blocking STAT7 or IL‐10 signaling led to a diminished bacterial load in the lungs of infected mice, yet without a significant alteration in their inflammatory response.^494^

**FIGURE 5:**
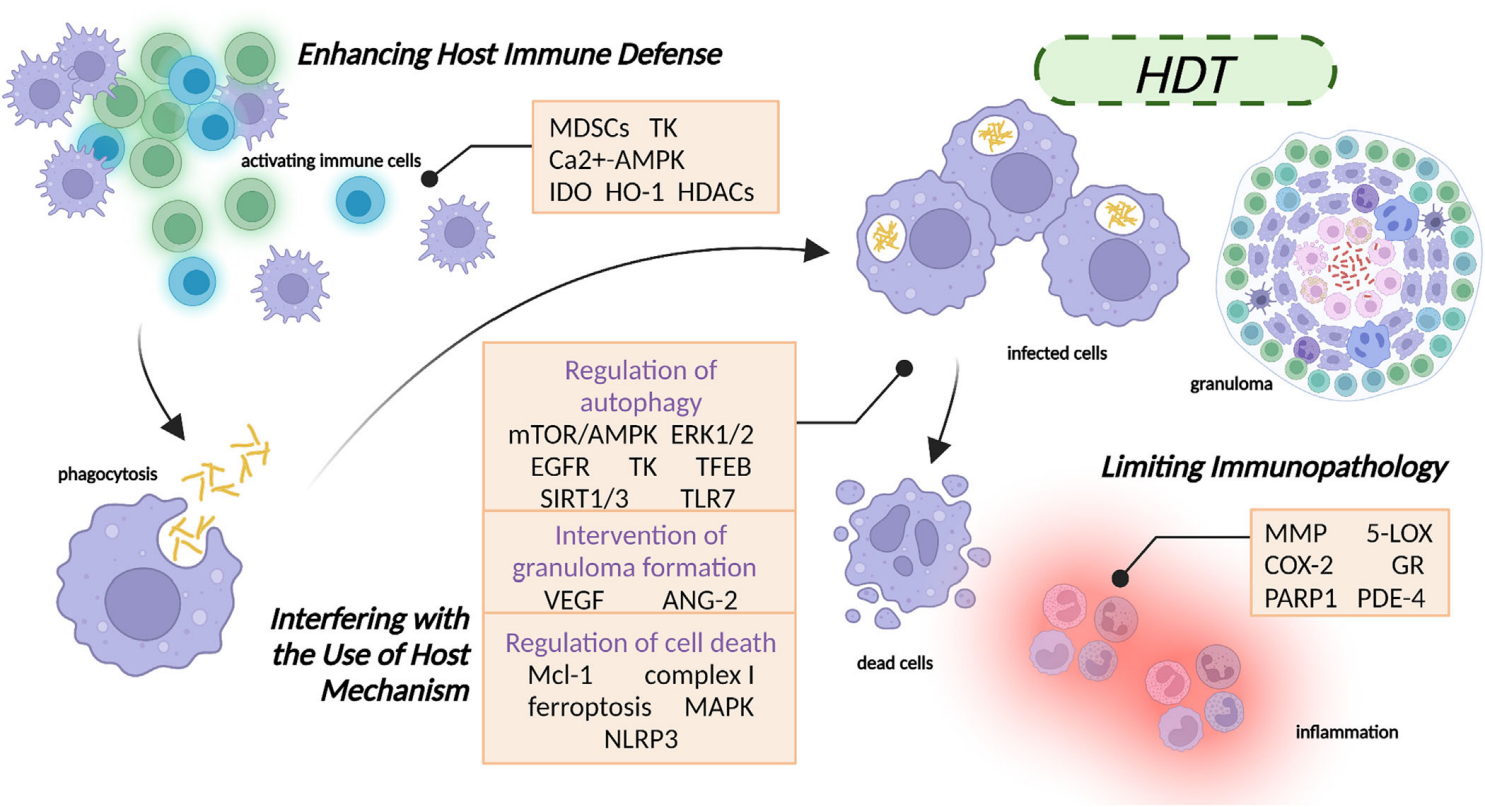
Overview of the host‐directed therapies (HDT) addressed. At the level of the host, targeting important processes such as immune defense, the use of host mechanism by *Mtb*, and inflammation regulation are invigorated to address and overcome drug resistance. The elements in the figure were drawn using BioRender online tool (https://biorender.com).

Figure 5 provides a comprehensive summary of the primary cellular processes and prospective targets implicated in HDT. The administration of HDT medications to enhance TB outcomes represents a propitious approach, particularly for MDR‐ and XDR‐TB. Despite this fact, the available clinical data regarding HDT are grossly insufficient. For the nascent HDT, forthcoming research must prioritize drug repositioning alongside validating efficacy. Due to the intricacy of the diverse cellular processes in the host and the inconsistency in response to the identical medication, HDT necessitates comprehensive analysis across multiple models to reveal the implications of novel treatment opportunities.

## 5 DISCUSSION AND PERSPECTIVE

Although TB is a malady that can generally be treated and even eradicated, the process often entails a long and financially demanding regimen. This underscores the urgent need for the discovery and development of novel therapeutic agents against TB. It is disappointing that the occurrence of drug‐resistant genes and multidrug‐resistant strains of *Mtb* is distressingly common. This not only complicates the treatment and management of TB but also poses a significant threat to global health.^495^ Consequently, any new therapeutic strategy must prioritize the circumvention of any crosstalk or interaction with first‐line drugs whilst simultaneously minimizing the risk of inducing resistance to existing therapies. Achieving this goal necessitates not only exhaustive characterization of current resistance mechanisms and the establishment of effective strategies to circumvent any potential cross‐resistance, but also the identification and validation of novel biological targets, and action mechanisms and potent lead compounds with novel scaffold to guide subsequent preclinical research and development initiatives.

Thanks to advances in pathogenomics, structure–function analysis, and systems biology, the endeavor of identifying targets in infectious agents has been accelerated and refined. Historically, disruption of crucial cellular pathways in *Mtb*, such as cell wall synthesis and assembly, protein synthesis, and energy metabolism, has been regarded as a potent strategy for combating TB. However, current research has shifted its focus to newly uncovered targets within *Mtb*, such as DNA gyrase, ATP synthase, DprE1, and MmpL3, which offer a promising alternative approach to subvert existing drug resistance mechanisms and have yielded remarkable outcomes. Structural determination of enzyme–ligand complexes lays the foundation for identifying key binding sites and designing high‐affinity therapeutics. Nonetheless, these drug candidates remain challenged in their ability to sufficiently engage both active and latent forms of *Mtb*, thus leaving behind a vicious cycle of recurrence.

As known, *Mtb* has coevolved with humans and has developed a remarkable degree of adaptation to the human host. Meanwhile, the latent form of the disease evades host immunity and retains the ability to cause disease upon reactivation. HDT seeks to achieve enhanced therapeutic outcomes and prognoses by judiciously manipulating host cellular processes. In healthy individuals, the foremost response upon exposure to *Mtb* is immunization: a cascade that begins with uptake of the pathogen by innate immune cells, which trigger the activation of adaptive immunity. Approaches aimed at optimizing this sequence represent an efficacious strategy for harnessing HDT. Interventions aimed at augmenting autophagy, dampening granuloma formation, and regulating host cell death to interfere with pathogen replication and retention have emerged as promising avenues for influencing the outcome of TB treatment. Active infection with *Mtb* elicits a potent immune response that often results in host‐mediated tissue damage. Combining other treatments with modulation of the inflammatory response to decrease tissue damage and cavitation can enhance the effectiveness of TB treatment.

In the context of comorbidities such as HIV infection or diabetes, HDT offers the opportunity to tailor TB management to each patient's unique clinical circumstances. Such patient‐specific tailoring of TB management cannot be readily achieved with antibiotics alone. Furthermore, the administration of combination HDT has consistently yielded improved therapeutic efficacy and abbreviated treatment durations across a diverse array of clinical trials. Additional benefits include the modulation of immunopathology and the facilitation of immune memory development. However, the toxicity potential of HDT must still be vigilantly monitored and actively assessed, and the combination strategies and underlying mechanisms of action must be subjected to rigorous scrutiny across multiple experimental models.

Undoubtedly, delving more deeply into the pathogenesis of TB will unveil additional targets for therapeutic intervention. These newly identified targets demand urgent attention and intensive investment to identify and optimize suitable drug candidates. For instance, target‐based virtual library HTS can be a potent and efficient method for expeditiously identifying leading compound candidates while substantially mitigating the time and cost of drug development. Drug repositioning strategies likewise hold great promise, as numerous US FDA‐approved drugs are currently under active investigation for their potential utility in the management of TB. It is clear that the successful control of TB represents a shared goal of both governments and civic society, and we must remain unwavering in their efforts to attain this objective.

## AUTHOR CONTRIBUTIONS

Y. L. and J. Y. conceived the study and wrote the paper. L. Z. revised the figures. Y. L. and W. Q. revised the article. All authors read and approved the final manuscript.

## CONFLICT OF INTEREST STATEMENT

The authors have no conflicts of interest to declare.

## ETHICS STATEMENT

Not applicable.

## Data Availability

Not applicable.
